# Harnessing ultrasound-responsive technologies for precision periodontitis therapy: a perspective

**DOI:** 10.7150/thno.138283

**Published:** 2026-09-28

**Authors:** Chao Xing, Sibtain Muhammad, Bing Guo

**Affiliations:** 1 Department of Stomatology, The People’s Hospital of Baoan Shenzhen, No. 118 Longjing Road, Baoan District, Shenzhen, Guangdong 518101, China.; 2 School of Science, College of Frontier Sciences, Harbin Institute of Technology, Shenzhen, 518055, China.

**Keywords:** periodontitis, sonodynamic therapy, piezoelectric dynamic therapy, bacteria, ultrasound

## Abstract

Periodontitis disease normally shows oxidative stress, immunological dysregulation, alteration of the periodontal bacterial ecology, and persistent alveolar bone loss. These mechanisms aggravate problems across the body in conjunction with causing tooth loss. The-state-of-the-art therapies using antimicrobial agents and mechanical debridement often fail to regulate immune signaling, clear infection, or repair tissue. Most recently, ultrasound-responsive materials have stood out to show advantages in restoring periodontal balance, which include two types (piezoelectric and sonodynamic materials). For sonodynamic materials, they generate reactive oxygen species (ROS) via acoustic cavitation, which degrades biofilms and disables microorganisms through oxidative damage. For piezoelectric materials, they convert mechanical stress into electrical polarization, resulting in surface charges that drive redox processes, affect immunological responses, and stimulate bone growth. Importantly, we highlight, to improve the periodontitis treatment outcomes, how these materials are incorporated into platforms such as injectable hydrogels, membranes, and scaffolds, which serve as carrier matrices for placing active medicines and improving spatiotemporal control. Also, we point out that integration of piezoelectric and sonodynamic technologies provides a way to combine antibacterial activity with regeneration stimuli. Moreover, challenges such as material stability, activation accuracy, and clinical translation are there and greater mechanistic understanding and rigorous design criteria are required for further development.

## 1. Introduction

Periodontitis is an inflammatory condition triggered by dental plaque, which gradually leads to tooth mobility and loss as the alveolar bone and periodontal ligament break down 1. Epidemiological data show that 800-1.4 billion people globally suffer from discomfort due to periodontitis and 23.6% of dentate adults have severe periodontitis 2. Severe periodontitis afflicted nearly 1 billion people in 2021 (1066.95 million; 95% UI: 896.55-1234.84), with a 12.50% (10.53-14.49) worldwide age-standardized prevalence. With a prevalence rate of 17.57% (14.73; 20.14), South Asia was at top among all. Globally, edentulism afflicted 353 million persons (300.60-416.20), having a 4.11% (3.50; 4.83) age-standardized prevalence. Latin America and the Caribbean have the greatest rates of edentulism (7.39% (6.44; 8.39) and by 2050, more than 660 million persons (+84.40%) will be edentulous and more than 1.5 billion (+44.32%) will have severe periodontitis. By that time, China will have 19.67% (130.23 million) of the global edentulous population. Severe periodontitis and edentulism are expected to increase by one and nine places, respectively, by 2050, in the list of the most important Level 4 diseases and disorders contributing to YLDs worldwide 3. In 2021, severe periodontitis and edentulism continue to be serious public health issues, and the number of afflicted individuals is expected to increase dramatically during the next decades 4. Edentulism and severe periodontitis are serious public health problems 5. Successful and effective public health interventions are urgently needed as prevention and control of these conditions at the population level remain a challenge **(Scheme 1)**.

Periodontitis is now recognized as a localized oral disease, but also a systemic inflammatory condition, involved in the pathogenesis of endocrine, cardiovascular, respiratory, autoimmune and neurodegenerative diseases 6. This link is mediated by migration of periodontal microbes and the persistence of inflammatory cytokines in the bloodstream 7. Periodontitis is an oral pathology comprising a persistent bacterial infection and altered immune response that results in a progressive loss of alveolar bone and supporting tissues of the tooth, culminating in tooth loss 8. Porphyromonas gingivalis is the most important pathogenic bacterium in the complex microbiome that drives initiation and progression of the disease through an imbalance in the relationship between the host and the microbiota 9.

Despite improvements in clinical care, current treatment strategies remain inadequate. Conventional non-surgical and surgical periodontal therapies primarily focus on mechanical cleaning and tissue regrowth, but they do not completely reconstruct the original periodontal system. Contrary to this, current biological systems treat tissue regeneration and antimicrobial activity as distinct objectives, neglecting the intimate relationship between inflammation and the process of bone creation. A major obstacle to long-term, successful periodontal care is this disparity 10. A hybrid approach that can both assist tissue repair and fight harmful bacteria is required. Because they are non-invasive and offer precise spatial and temporal control, phototherapy techniques like photodynamic therapy (PDT) and photothermal treatment (PTT) offer a promising path ahead 11. But in its present state, PTT usually kills bacteria when the temperature rises beyond 50°C, which puts the surrounding tissue at danger. The importance of low-temperature PTT techniques that effectively eradicate germs with little tissue injury is emphasised in this research 12.

PDT, on the other hand, has a photochemical effect in which light-activated photosensitisers produce ROS, including singlet oxygen species, which damage bacterial membranes and biofilms 13. This increases the susceptibility of bacteria to death and, when combined with heat, produces a potent bacterial killing impact at lower temperatures. For phototherapy, light absorption is essential. Deep periodontal infections cannot be treated with the conventional near-infrared window (NIR-I, 650-900 nm) light, despite its modest light penetration 14.

However, the second near-infrared window (NIR-II, 900-1700 nm) exhibits deeper penetration, lower scattering and higher permissible power, which can be used for deep tissues 15. Thus, combining low-temperature PTT and PDT with NIR-II-responsive nanomaterials holds the potential for advanced periodontal therapy. Such approaches could potentially solve the problems of existing approaches by facilitating targeted killing of bacteria while also promoting tissue regeneration, which ultimately address both the microbial and structural disease components 16.

In clinic, Photodynamic therapy for oral cancer and oral bacterial killing is popular, because of most are exposed outside with accessibility by light 17. However, light cannot penetrate deep inside some oral disease tissues. To move forward, ultrasound is applicable, in which sonodynamic sensitizers and piezoelectric materials are available to be customized for treatment of oral diseases like periodontitis, especially bacterial, wound, bone defects, and even cancer 18. Rather than electromagnetic waves, Ultrasound (US) is acoustic and mechanical waves with a frequency above the human audible range (16-20 kHz). Low-frequency (1-3 MHz) ultrasound has a penetration depth of 10 cm with acoustic pressure pulses, whereas phototherapy is limited by the penetration depth (<1 cm) 19. Ultrasound results from the effects of acoustic chemistry and acoustic-mechanical effects. It causes thermal effects due to physico-mechanical vibration (ultrasonic cavitation) and promotes ROS formation by acoustically sensitive substances. One new non-invasive antimicrobial treatment approach is sonodynamic therapy (SDT) 20.

Early ideas attributed the cytotoxicity of SDT to the production of singlet oxygen (1O_2_), however the mechanism underlying SDT is still up for debate. But as study developed, researchers suggested that the cavitation phenomena caused by ultrasound is the basis for the biological impacts of SDT. Cavitation is the process by which ultrasonic activity causes microbubbles to develop at the medium's source. Only stable cavitation is produced by low-intensity ultrasonic stimulation, which does not jeopardize the integrity of the cell membrane and, hence, cell function 21. SDT affects cell activity by reducing the energy threshold needed for cell membrane mechanical damage during ultrasonic stimulation, but only when combined with sonosensitizers. Sonosensitizers, ultrasound, and oxygen are the three essential elements of SDT 22.

Piezoelectric materials are a subgroup of sonosensitizers that show ferroelectricity and/or piezoelectricity 23. Black Phosphorus (BP), zinc oxide (ZnO), barium titanate (BaTiO_3_), and so forth are examples. When piezoelectric materials are used as sonosensitizers, ultrasonic stimulation causes electron-hole (e-h+) pairs to separate inside the materials, creating an internal electric field 24. Through redox reactions, the resulting electrons and holes go to their opposing surfaces, generating a significant amount of ROS 25. US based a group of different materials have been summarized for periodontitis 26. Yang *et al*. 27 recently investigated how SDT using nanomaterials can be used to treat tumors. SDT is expected to have a big impact on cancer therapy in the future by providing patients with more effective and personalized treatments. Suk *et al*. 28 showed that placement of a piezoelectric dermal patch on the wound region may result in the formation of an EF to accelerate the wound healing process during deformation due to animal's movement. Wu *et al.* 29 devised piezoelectric nanocomposites (NCs) by attaching gold nanoparticles to barium titanate (BTO) nanocubes. The authors observed that the piezoelectric effect of the NC may induce the generation of ROS to kill bacteria under US stimulation. But the antibacterial effect of piezoelectric materials is not good enough to replace antibiotics. Thus, there still needs to be new and better therapeutic methods developed to optimize treatment for antibacterial and wound healing therapies. Zhu *et al*. 30 thus developed a strategy to control the generation of ROS during the wound healing process using MOFs and piezoelectric nanomaterials. This approach successfully achieved a proper balance between the antibacterial and repair-promoting actions and provided new insights into the design of an effective multifunctional system for biomedical applications. Moreover, Yao *et al*. 31 examined piezoelectric materials, nanogenerators, zwitterionic hydrogels, triboelectric scaffolds, biological effects, processes, and applications for bone and cartilage tissue engineering in a methodical manner. Currently, Hydrogel microspheres (HMs) have gained more interest as hydrogel technology has developed because of its numerous biological uses. Huang *et al*. 32 summarized the uses of HMs in wound healing, tumor immunity, regenerative medicine, drug delivery, and cell culture has enormous potential to advance biomedical treatments.

The recent literature illustrates a clear shift toward multifunctional, stimulus-responsive platforms for oral infection control and periodontal regeneration. In order to overcome the low catalytic efficiency of traditional piezoelectric systems, Zhou *et al*. 33 employed heterojunction construction to create a piezoelectric hydrogel that combined antibacterial activity and periodontal tissue healing with enhanced charge separation and ROS production. Xing *et al*. 34 created a piezocatalytic platform that combines teeth whitening with antibacterial activity and microbial environment management in a different oral application by using Sr/Ca co-doping to alter the physicochemical behaviour of BaTiO_3_. Medicinal uses have also been made of the mechanical forces produced during regular mouth activity. In order to prevent bacterial colonization at the peri-implant interface, Chen *et al*. 35 created an occlusion-responsive piezoelectric implant that transforms typical physiological stress into local electrical stimulation.

In a distinct work, Chen *et al*. 36 reported a Janus piezoelectric membrane that provides both physical shielding and regenerative regulation of the periodontal microenvironment by combining asymmetric barrier protection with disease-responsive bioactivity. Liu *et al*. 37 showed that magnetoelectric stimulation can change macrophage behaviour and foster a regenerative immunological environment in sick periodontal tissue, going beyond direct antibacterial activity. Yin *et al*. 38 shown a multidisciplinary energy exchange, which combines LSPR with piezo-phototronic effects to enhance oral sterilization and teeth whitening. Zhang *et al*. 39 created an ultrasound-activated, dual-layer piezoelectric bionic periosteum for hard-tissue repair with the goal of simulating key elements of the periosteal-bone interface and facilitating bone regeneration. Du *et al*. 40 investigated complementing breakthroughs in precision nanomedicine, focusing on bio-nano structures capable of coordinating antimicrobial delivery, immunological regulation, and disease microenvironment remodeling during periodontal regeneration. Moreover, Wang *et al*. 41 studied the coupling of piezoelectric materials with bone organoids, establishing a new framework for studying mechanically driven electrical signaling and assessing regeneration responses in more physiologically appropriate three-dimensional situations. Collectively, these discoveries show that the field is evolving above single-function antimicrobial materials and toward adaptive platforms that combine externally or physiologically produced physical signals with microbial control, immunological management, and structural tissue repair.

Integrating new material platforms with ultrasound-responsive and mechanically activated features offers a promising path forward for periodontitis therapy, especially for addressing the disease's multifaceted pattern, including infection, chronic inflammation, and bone loss over time. Recent advancements in technology, including as injectable hydrogels, bioadhesives, Janus membranes, implant coatings, and nanoscale carriers, have offered a number of platforms for targeted, time-controlled medicinal delivery inside the periodontal cavity 42. Prospective therapies that are self-powered and remotely controlled will be made possible by combining ultrasound-responsive therapy with piezoelectric or triboelectric components 43. In order to provide temporally and spatially controlled electrical stimuli and ROS events for on-demand antimicrobial delivery, immunological regulation, and osteogenesis, these methods may combine endogenous mechanical stimuli (such as chewing and swallowing) with external ultrasound 44. For instance, including piezoelectric components into dental restorations and implants might provide ongoing mechano-electric stimulation, which would improve osseointegration and decrease in-situ biofilm 45 **(Scheme 2)**.

Implementing these concepts requires consistent performance under repeated mechanical pressure, long-term stability in the saliva-rich oral environment, and precise regulation over the activation level. This analysis balances the potential and limitations of these devices for treating periodontitis by examining their design principles, methods of action, material types, and biological effects. The goal is to reduce the time between the discovery of novel materials and dental applications that are therapeutically beneficial.

## 2. Ultrasound-responsive materials

Usage in catalysis, conventional imaging, and treatment are made possible by the emergence of ultrasound-responsive materials, a developing class of stimuli-responsive systems that may transform sonic energy into chemical and electrical outputs 46. Sonodynamic and piezoelectric materials are the two main platforms in this field that have garnered a lot of interest. Inorganic semiconductors like TiO_2_ and ZnO, organic systems like porphyrins and chlorins, and hybrid platforms like metal-organic frameworks and carbon nanomaterials are examples of sonodynamic materials, often referred to as sonosensitizers (47). During ultrasound, cavitation and sonoluminescence activate these materials, creating localized high-energy conditions that encourage electron excitation and, consequently, the production of ROS. Ceramics like BaTiO_3_, PZT, and ZnO, polymers like PVDF and P(VDF-TrFE), and new nanostructures like boron nitride and two-dimensional materials are examples of piezoelectric substances 48. These tools facilitate electrochemical activity and bioelectrical stimulation by converting mechanical strain into electrical polarization. The processes of piezoelectric and sonodynamic materials are essentially different 49.

Sonodynamic devices provide brief bursts of high temperature and pressure via sonic cavitation, which encourage the generation of radicals and ROS-mediated effects 50. Piezoelectric materials operate differently, generating charge by lattice-strain-induced charge shift described by Energy Band Theory, which encourages redox reactions and surface polarization. According to the Screening Effect, these charges are quickly filtered out under physiological conditions as a result of ion compensation and electro-interface reconstruction 51. This disparity shows that although piezoelectric systems priorities electromechanical coupling and charge separation, sonodynamic systems concentrate on ROS formation. Through stiff bulk ceramics with high electromechanical coefficients to flexible polymers, nanoscale fillers, and hybrid composites with enhanced biocompatibility and adjustable properties, piezoelectric materials have made advances (Table 1). To increase energy conversion efficiency and biological performance, recent developments have focused on surface functionalization, nanoscale engineering, and integration with other responsive systems 52. However, challenges remain in achieving accurate activation, sustaining long-term stability, and guaranteeing reproducible behaviour in complex circumstances, underscoring the necessity of design solutions based on enhanced mechanistic understanding and translational promise 53.

### 2.1 Inorganic piezoelectric materials

Inorganic piezoelectric materials can be broadly divided into single crystals and polycrystalline ceramics, with different structures and properties 54. Single-crystal piezoelectric materials have long-range ordered domains, while piezoelectric ceramics are polycrystalline with crystallites randomly oriented, which must be poled to induce dipoles and result in a macroscopically piezoelectric material 55. Recently, material development has advanced from single-component systems, like BaTiO_3_ and PbTiO_3_, to more complex compositions near morphotropic phase boundaries (MPBs), such as lead zirconate titanate (PZT), (1-x)Pb(Mg_1_/_3_Nb_2_/_3_)O_3_-xPbTiO_3_ (PMN-PT), and Ba(Zr0.2,Ti0.8) O_3_-x(Ba0.7, Ca0.3)TiO_3_ (BCT-BZT). The MPB compositions display superior electromechanical properties associated with the phase co-existence and the flexibility for polarization change 56,57. Recently, techniques such as heterovalent doping have been used to modulate the electrical and structural properties to further enhance the performance of piezoelectric ceramics, thereby leading to a newer trend in material development 58.

The majority of high-performance piezoelectric materials have a perovskite crystal structure ABO_3_, where the A-site cations are usually alkaline earth or rare-earth cations and the B-site cations are transition metals 64. PZT has long been domineering for its piezoelectric performance, although its application in biomedical devices is greatly restricted by the toxic element lead. As a result, the need for lead-free alternatives, with both technical performance and biocompatibility, has been promoted 65. One of the most well-studied lead-free piezoelectric materials is barium titanate (BaTiO_3_, BTO) due to its excellent biocompatibility and large electromechanical coupling. Spontaneous polarization in its non-centrosymmetric tetragonal phase results in strong piezoelectric and ferroelectric properties 59. In its nanoscale form, the increased surface activity of BTO enables it to be used in applications such as piezocatalysis, antibacterial treatment, and dental tissue engineering. Alternatively, the lead-free potassium sodium niobate (KNN) system exhibits relatively high piezoelectric coefficients, good thermal stability and good mechanical properties, making it a suitable substitute for lead-based materials 66.

Beyond perovskite oxides, single-layered materials like Bi_2_WO_6_ have been explored as catalysts that are coupled with piezoelectric properties. The anisotropic nature of Bi_2_WO_6_ can transform it into a mechanocatalyst under such stimulation to release reactive species for killing bacteria and eradicating biofilm. There is also emerging evidence of their use in cancer treatment, but this is still in its infancy 67. While inorganic piezoelectric ceramics have several benefits - such as high sensitivity, stability and powerful piezoelectricity - they also have drawbacks. Their brittle nature and susceptibility to operating conditions (including temperature, frequency) limit their applications, especially in flexible and dynamic biological environments. These issues have driven an interest in organic and hybrid piezoelectric materials with better flexibility and deformability, but generally at higher piezoelectric losses 68.

### 2.2 Organic piezoelectric materials

Organic piezoelectric materials are currently drawing much attention for use in biomedical applications, as they are generally non-toxic, flexible, processable and stable. But polymer-based piezoelectrics also have some inherent drawbacks, including low piezoelectric coefficients and poor mechanical properties 69. One of the most studied examples of organic piezoelectric materials is the polymer poly(vinylidene fluoride) (PVDF), in which the repeat unit has a head (CH_2_) - tail (CF_2_) structure. PVDF can exhibit five polymorphic phases (α, β, γ, δ and ε) 70. The β-phase is the most relevant for piezoelectric activity as it orients all of the dipole moments in a single direction to give the highest dipole moment (reported up to 2.1 D) as a result of the highly polar C-F bonds 71. Wang *et al*. 72 developed another copolymer called poly(vinylidene fluoride-trifluoroethylene) (P(VDF-TrFE)) for use in flexible sensors, tissue engineering and regenerative medicine. Researchers have shown that nanogenerators based on electrospun nanofibers of P(VDF-TrFE) can provide local electrical stimulation, resulting in improved proliferation of preosteoblast cells in culture.

Ico *et al.* 73 proposed that electrospinning can enhance the piezoelectricity of the material through the adjustment of fiber diameter and molecular orientation. Power sources outside of PVDF systems can also generate electricity through the use of other organic polymers that are modified to include piezoelectric properties through structure modification or treatment. For instance, electrical poling, which involves applying a high voltage to permanently orient dipoles, can be used. For instance, poly(tetrafluoroethylene) (PTFE) can be poled into a piezoelectric electret capable of producing electrical responses as a result of mechanical strain 74. Notably, PTFE can also generate ROS as a result of its electromechanical behaviour and this may have potential in chemical and therapeutic applications 75.

For instance, in nylon-11, the antisolvent crystallization process may result in highly ordered nanoparticles that can contain highly oriented dipoles. This increased order leads to greater mechanical stability and energy localization in the material 76. Natural biodegradable polymer polylactide (PLA) is also a significant piezoelectric material. It is piezoelectric both in the crystalline and amorphous states (though the coefficient is typically smaller than conventional piezoelectric polymers). This is due to its chiral structure in which the polymer chain is in a helical conformation that includes polar ester (–CO-O-) groups bound to asymmetric carbon atoms. Putting a strain across the chain axis can somewhat twist these dipoles, leading to in a net change in the polarization that is parallel to the straining axis, and consequently creating a net electric energy potential 77. Schönlein *et al*. 78 demonstrated that improvements to the piezoelectric efficiency of PLA can be accomplished by enhancing its optical reactivity and by post-stretch annealing. Particularly, poly(L-lactic acid) (PLLA), which is made up of L-isomers, has been to exhibit a structure-dependent piezoelectrical response while crystallographic state and molecular orientation can have substantial effects on its electromechanical responses. Poly(3-hydroxybutyrate) (PHB), which corresponds to the group of polyhydroxyalkanoates (PHA), is also a biodegradable piezoelectric polymer that can be utilized a for biological applications. Vatlin *et al*. 79 discovered that activated PHB films not only display piezoresponse but additionally exhibit antibacterial activity. Furthermore, PHB coupled with chitosan (CS) has been demonstrated to increase electrostrictive properties, hence increasing the piezoelectric coefficient. The CS-PHB films have not merely greater biocompatibility, but also stronger piezoelectric capabilities than PHB, which offers enormous potential in the field of tissue regeneration and regenerative therapies.

### 2.3 Piezoelectric composites

Piezoelectric systems comprised of one component (such as ceramics or polymers) are widely used, although they may not satisfy all of the criteria for medical uses 60. To address these issues, multifunctional piezoelectric materials were designed. This maintains the advantages of piezoelectric devices while combating the disadvantages of their components (such as the fragile nature of inorganic ceramics). Hence composite systems generally demonstrate superior electromechanical coupling, greater flexibility and increased biocompatibility 61.

Piezoelectric composites can be tailored in various physical forms such as fibers, films and hydrogels, to suit their expected applications. These composites, though highly promising, face a number of obstacles in their applications. These include complex synthesis processes, challenges in optimizing mechanical properties and piezoelectric performance, and poor phase compatibility 80. Piezoelectric composites for dental and biomedical applications can be broadly grouped into several groups with different compositions. These include, but are not limited to, inorganic piezoelectric materials combined with organic piezoelectric polymers, inorganic phases mixed with biodegradable polymers, hydrogel piezoelectric composites, metal-piezoelectric composite, and other multi-phase/piezoelectric composites 81. For instance, composites based on BaTiO_3_ nanoparticles and P(VDF-TrFE) are reported to enhance the generation of piezoelectric charge for bone regeneration. Likewise, BaTiO_3_ or ZnO based piezoelectric materials composites with polymers such as PVDF or P(VDF-TrFE) are also extensively explored 82. Here, the inorganic material is generally responsible for the piezoelectric response, with the polymer matrix offering flexibility and mechanical support. However, it is important to achieve homogenous filler distribution and effective bonding between filler and matrix since these contribute significantly to the properties of the composite. Other factors such as particle polarization and structural stability of the inorganic fillers are also critical 83.

The use of biodegradable polymers instead of synthetic polymers provides further benefits to the composite. They are degradable by microorganisms to environmentally friendly carbon dioxide and methane. As a result, inorganic - polymer piezoelectric composites with biodegradable polymers are sought for their biocompatibility, non-toxic degradation products and potential for antibacterial activity 84. Metals can also be added in the inorganic/polymer composites to enhance electrical and mechanical properties for better performance in more novel medical applications. Another important group are piezoelectric hydrogels, which have increasing applications for wound dressings and drug delivery. These hydrogels have antibiotic, anticancer and antioxidant properties which are highly suitable for drug delivery in the medical field, such as in regeneration and cancer treatment 85. However, conventional hydrogels can suffer from poor drug release, stability and/or dispersity *in vivo*. One potential solution is to use drug-free piezoelectric hydrogels. When mechanical stress is applied, these materials release bioelectric signals, producing a self-regulated effect that may help avoid producing drug resistance 86.

In summary, hybrid piezoelectric composites - such as those based on polyhydroxyalkanoates (PHA) or lead-free PVDF and P(VDF-TrFE) systems - synergistically combine the high electromechanical properties of inorganic piezoelectric ceramics with the softness and biocompatibility of polymers. Such unique characteristics render piezoelectric composites very promising for sophisticated dentistry and medicine.

## 3. Mechanistic study of ultrasound responsive treatment of periodontitis

Plaque biofilm-induced periodontitis is the major cause of adult tooth loss and alveolar bone destruction 87. Periodontitis, also the sixth most prevalent infectious disease globally, is also related to systemic health and increases the risk for Alzheimer's disease, cardiovascular disease, diabetes, and certain cancers 88. Consequently, appropriate periodontal treatment is not only important for oral health, but also for overall health. The current clinical treatment of periodontitis is largely centred on mechanical debridement, surgery and antibiotics, which have their limitations. Mechanical debridement has low efficacy to access and remove plaque biofilm from deep periodontal pockets. Surgery is invasive and may be painful. Antibiotic use risks the development of resistance and can cause gut issues. Due to the above limitations, new effective and non-invasive treatment options in periodontology are needed 89.

Ultrasound-responsive treatment techniques have emerged as promising approaches to periodontitis care because they convert external mechanical energy into biologically active signals. Although applying ultrasound as a stimulus, SDT, piezodynamic treatment (PZDT), piezocatalysis, and piezoelectric bioelectrical activation all operate via essentially distinct procedures, resulting in a variety of therapeutic results. SDT primarily creates ROS by activating sonosensitizers, whereas PZDT produces ROS and localised charge via ultrasound-induced piezoelectric polarisation 90,91. Piezocatalysis, on the other hand, employs piezoelectric surface redox reactions to sustain ROS-mediated antibacterial effects, whilst piezoelectric bioelectrical activation produces electrical signals that govern cellular behaviour and stimulate tissue repair. Because these processes differ significantly, differentiating between the two is vital for understanding their advantages and roles in periodontal therapy 92-94. **Table 2** illustrates the energy inputs, physicochemical mechanisms, therapeutic outputs, and biological repercussions of periodontitis treatment and regeneration.

The effectiveness and safety of ultrasound-responsive periodontal treatments are determined by the entire acoustic dosage, which includes frequency, intensity, power density, duty cycle, exposure duration, and treatment recurrence. In excess, ultrasound can generate heat buildup, excessive cavitation, uncontrolled ROS formation, quicker material degradation, and excessive ion release; inadequate amounts might not be enough to produce any effect. Therefore, ultrasound parameters should be optimized alongside temperature elevation, ROS generation, ion-release kinetics, host-cell tolerance, and material retention to establish a defined therapeutic window for safe clinical translation.

SDT is an alternative non-invasive treatment. It's a method of US-induced activation of sonosensitizers, which induces ROS and mechanical damage to kill bacteria. SDT is highly biocompatible, has good penetration and is highly accurate, and thus has the potential to treat biofilm infections at a deeper level. Moreover, given the widespread use of ultrasonic cleaning in dentistry, SDT is a promising, readily implementable treatment for periodontitis. SDT is dependent on sonosensitizers. But its effectiveness is severely limited by the thick extracellular polymeric substances (EPS) present in biofilms, which act as a barrier, limiting the penetration and interaction of sonosensitizers and attached bacteria. This directly affects the generation of sufficient ROS, preventing its antibiofilm activity 95. To overcome this limitation, sonosensitizers have been modified with positively charged molecules **(Scheme 3)** 96. These positive modifications enable the agents to enter into the negatively charged bacterial cell walls, which increases the vulnerability of the germs to sonodynamic attacks. So, the use of positive-negative charge interactions is a way to enhance the penetration and targeting ability of biofilms.

Periodontitis is partly caused by reciprocally reinforced interactions between the host inflammatory response and the dysbiotic microbioms 97. Periodontitis has been repeatedly associated with a variety of systemic conditions, such as cardiovascular diseases, type 2 diabetes, obesity, rheumatoid arthritis, osteoporosis, respiratory tract infections, inflammatory bowel disease, Alzheimer's disease, non-alcoholic fatty liver disease, chronic kidney disease and various cancers **(Figure 1A)** 98. An important research question is whether the links are simply correlative or causal, as this has strong implications for patient care and treatment strategies. A popular theory holds that low-grade systemic inflammation originates in the periodontal tissue. This chronic inflammatory state is considered to represent a common biochemical mechanism that connects periodontitis to a variety of inflammatory comorbidities. Meanwhile, systemic diseases might have an adverse effect on dental tissues.

For example, in type 2 diabetes, elevated inflammatory burden and alterations in the oral microbiome can significantly aggravate periodontal tissue destruction.

Periodontitis is thought to exert effects on systemic health, and this is thought to occur via several mechanisms. These include the passage of periodontal microorganisms into the circulation, and also the spread of inflammatory cytokines from the periodontal tissues into the bloodstream. While most studies have researchers examined bacterial involvement, the oral microbiome includes viruses and other microbes that may modulate the host immune response and may act in concert with the bacteria. The trafficking of immune cells is another hypothesis. Stimulated lymphocytes from periodontal draining lymph nodes may travel to distant tissues via the lymphatics or the blood stream, where they may intensify inflammatory reactions. Finally, the inflammatory response of periodontitis may lead to sustained changes in hematopoietic stem and progenitor cells in the bone marrow. This process, also known as "trained myelopoiesis", results in the generation of pro-inflammatory myeloid cells, and may increase the risk of systemic diseases **(Figure 1B)**. Spread oral microorganisms may also directly affect various organs. They have been shown to cause lung infection, to alter vascular function, and have even been involved in tumor development. Specifically, as with many diseases, several species have been linked to different cancers such as colorectal cancer and upper digestive and oral squamous cell carcinomas.

Importantly, clinical intervention studies have shown that effective periodontal therapy can reduce systemic inflammatory markers and improve surrogate indicators of related diseases. These studies confirm the notion that periodontitis is a risk factor for systemic disease that can be modified. Consequently, there is now consideration in the adjunct use of host-modulation therapies to conventional periodontitis treatment. For instance, complement pathway inhibition has already demonstrated a positive effect in initial clinical studies of periodontal inflammation. In conclusion, the link between periodontitis and systemic disease is a bi-directional multifaceted inter-relationship between microorganisms, inflammation and immunology. Such a relationship underlines the need for oral and systemic health, including the health of the periodontium.

## 4. Sonodynamic sensitizer-based therapy for bacterial killing

Antibiotic therapy for bacterial infections caused by biofilms is often ineffective and leads to medication resistance 99. US-responsive antibacterial systems have demonstrated promising potential to eradicate bacterial biofilm through stimulating distinct sonophysical and sonochemical effects. By using US-triggered nanotherapeutic approaches producing reactive oxygen species (ROS), in particular, innovative systems are being developed to combat multidrug-resistant bacterial infections, while promoting tissue repair 100. One such strategy is non-invasive SDT. This technique involves the activation of sonosensitizers by ultrasonic waves, resulting in the production of toxic ROS via controlled cavitation, mechanical and thermal effects. These are known to damage microbial structures and cell functionalities. Sonosensitizers can be classed as organic or inorganic. Examples of inorganic sonosensitizers include metal oxides, metal carbides, metal hydrides and metal-organic frameworks (MOFs), which are under active development for SDT clinical studies 101. Yet concerns remain around their long-term safety, namely poor clearance and unwanted tissue accumulation. Organic sonosensitizers, in comparison, provide higher adaptability, biocompatibility, and adaptability.

However, their applications are hindered by issues related to their instability in biological media, quick systemic clearance, and phototoxicity. Thus, significant research has focused on designing hybrid systems and tailored sonosensitizers incorporating the advantages of both organic and inorganic materials to enhance their efficiency and stability 101. Furthermore, the engineering of nanobiocatalytic systems has boosted SDT. Smartly engineered nanomaterials can enhance ultrasound-induced ROS production, enhancing the separation of charge carriers, and higher ROS yields on sonication. Moreover, some of these systems display multi-functionality with intrinsic catalytic activity synergistically acting with ROS generated from ultrasound exposure to produce a "self-amplifying" antimicrobial effect. This facilitates better control of oxidative environment. Furthermore, in wound healing, endogenous ROS such as hydrogen peroxide can be employed by catalase-like nanozymes to produce oxygen. This not only promotes bacterial killing, but also retains oxygen for angiogenesis and repair processes 102.

Taking inspiration from the iron-dependent peroxidase enzymes that naturally exist in living organisms, Zhao *et al*. 103 developed a metalloporphyrin-based nanozyme, poly(osmium porphyrin) (Polypor (Os)), to synergistically treat drug-resistant infections under conditions of hypoxia via ultrasound-based ROS generation. Thorough physicochemical characterization confirmed that Polypor (Os) combines intrinsic peroxidase-like activity with ultrasound activation to achieve spatial-temporal control of ROS amplification in a hypoxic infection site. *In vitro* tests confirmed that Polypor (Os) triggered by ultrasound quickly kills bacteria by compromising membrane integrity and interfering with metabolism through reactive oxygen species (ROS) production, with high cytocompatibility. Experiments done *in vivo* also validated its therapeutic performance, yielding successful infection eradication, improved angiogenesis, and decreased inflammation. In summary, this study has developed a safe and effective strategy for treating challenging infected wounds through incorporating physical ultrasound stimulation and efficient catalytic nanomedicine **(Figure 2A-B).**

Dental caries are another clinical infection of biofilm disease. These form through the gradual deterioration of the tooth surface, and often start at the surface layers of the enamel. While caries development can be attributed to a variety of factors, a key factor is the shift in bacterial composition in the biofilm, such as prevalence of acidogenic bacteria (such as Streptococcus mutans) in combination with poor oral hygiene. This alteration consequently results in deterioration of hard dental tissues. Thus, satisfied disruption or reduction of bioadhered bacteria is an integral factor in the prevention, cure of dental caries as well as secondary complications.

Recently, sonodynamic antimicrobial chemotherapy (SACT) has been proposed to treat infectious diseases. It involves the application of low-intensity ultrasound in conjunction with sonosensitizers (chemicals). It shares some similarities with antimicrobial photodynamic therapy (aPDT) in that low-frequency (Hz) sound waves are used, rather than light, and sonosensitizers instead of photosensitizers.

Another key concept here is that of the "bystander bioeffect", which refers to intercellular communication between treated and untreated cells. This effect can be direct or indirect, resulting in functional changes to other cells and, potentially, immune responses. In photodynamic therapy, this effect has been reported to amplify cytotoxicity in photodynamic therapy beyond directly irradiated cells, which may be due to communication between cells in biofilms, and the exchange of signaling molecules or microbicidal products. Several studies have investigated this effect in SACT.

For instance, Maryam *et al*. 104 explored bystander effects in Streptococcus mutans after sonocatalytic therapy using curcumin loaded poly(lactic-co-glycolic acid) nanoparticles (Cur-PLGA-NPs) as sonosensitizers. In their work, the proposed SACT-induced bystander effects on human gingival fibroblast (HGF) cells (which represent non-target or off-target cells) is shown in **Figure 2C**.

The development of ultrasound-responsive techniques like SDT, which use ROS production for antibacterial activity and tissue regeneration, was prompted by the fact that antibiotic medicines are still mainly ineffective against biofilm-associated illnesses. Inorganic and organic sonosensitizers are promising, but issues relating to toxicity, instability and fast elimination hinder their use in practice and suggest the need for more potent and biocompatible structures. While new progress in nano-biocatalytic platforms and hybrid systems has enabled higher efficiency on ROS generation and synergistic antimicrobial effects, there remains a lack of mechanistic understanding, particularly under natural and complex biofilm environments. In addition, the integration of such technologies within the unique oral microenvironment is not yet fully understood and concepts such as bystander effects are often extrapolated without specific validation for ultrasound-based approaches, although they are increasingly being investigated for effects such as cavitation and/or cavitation-like effects in biological systems as in dental infections such as caries by Streptococcus mutans. To make SDT more than a promising platform and rather a reliable treatment, such work needs to be complemented by greater mechanistic understanding, testing and models suitable for clinical applications.

## 5. Piezodynamic therapy for bacteria killing and wound healing

Bacterial infections have emerged as a major global threat to human health and well-being. Expanding data has revealed that microorganisms can indirectly contribute to the onset and advancement of various illnesses, including cancer 105. Thus, in the fight against bacterial illness, an antibacterial agent with high killing efficacy and shelf-life is constantly sought. Recently, photon energy was used to destroy bacteria using photocatalysis. Photocatalysts absorb photon energy and create electrons and holes, leading to the generation of active species including superoxide ions (.O_2_^-^) and hydroxyl radicals (.OH) 106. These, in turn, can destroy microorganisms. Despite widespread coverage of photocatalysis, practical applications of photocatalytic dye degradation are restricted due to expensive costs, lack of reactivity in the absence of light, low photo-conversion efficacy, and charge recombination 107.

In contrast, there has been an increased interest in utilizing the piezoelectric effect to destroy microorganisms. Piezoelectric materials are often used in sensors, transducers, water treatment, and actuators because they generate electricity when physically deformed. In the so-called piezocatalysis process, piezoelectric materials use mechanical energy to generate a significant number of electrons and holes, which can mix with oxygen and water and release free radicals to kill bacteria 108. Mechanical energy may come from several sources, such as wind, river movement, friction, and human action. Furthermore, when the charge carrier separation causes the piezoelectric materials to strain, a piezo potential develops. In general, the piezoelectric effect might be used in two ways to destroy germs 109. The piezoelectric potential might be utilized to improve photocatalytic efficiency by increasing the dissociation of photogenerated electrons and holes. It can also function as an independent source of energy to eliminate germs by creating ROS in response to mechanical stress. Sharma *et al*. 110 found that piezoelectric NaNbO_3_ nanorods inhibited E. coli with 23.0 mm inhibitory zones when combined with bacteria and subjected to mechanical vibrations.

Montoya *et al*. 111 added BaTiO_3_ into composite dental resins as an antibacterial agent. Wu *et al*. **112** also designed Au@BaTiO_3_ nanocomposites and found that they exhibited robust antibacterial activity against Gram-positive and Gram-negative bacteria under ultrasound. In recent years, researchers have focused on design and function, as well as degradability, simplicity of piezoelectric systems and stability.

Huang *et al*. 113 developed a Janus piezoelectric patch to achieve these objectives. The top layer of the patch was made of a PEGDA hydrogel matrix incorporating Au-decorated BaTiO_3_ nanoparticles for piezoelectric catalysis, while the bottom layer of the patch was made of a GelMA matrix incorporating VEGF to promote tissue regeneration. The evaluation of patches was done by treating wound sites infected with Staphylococcus aureus bacteria in rats, and using them in combination with ultrasound at 1.5 W cm^-2^ for 3 minutes per day for three days. Findings indicated that this system could endorse both the antibacterial effect and tissue regeneration synergistically under the stimulation of ultrasound.

On the other hand, a few kinds of multifunctional piezoelectric hydrogel patches have also been developed. One study developed injectable conductive hydrogels that generate electrical signals though converting the mechanical energy from body motions to speed up wound healing 114. Other studies employed asymmetrical hydrogels that allowed repetitive movements of wounds to be used for real-time monitoring and for providing antibacterial properties. Ultrasound technologies have also shown promise to provide better external control of the therapeutic effects. For instance, ultrasound-sensitive nanocomposite hydrogels can be activated by an external common stimulation to produce antibacterial and healing effects 115.

Other studies have also attempted to obtain piezodynamic and chemodynamic therapy (PT-CT) to combine the effects of antibacterial activity and immune regulation after ultrasound activation through the use of Cu^2+^/Zn^2+^ co-doped BaTiO_3_. On the other hand, piezoelectric tough dual-network hydrogels have been used to treat infected wounds, with the sonopiezoelectric effect aiding in regulating key signaling pathways 116. While these studies are promising, most of the current piezoelectric systems remain monopolar or passive. Although some recent works have begun to understand the signaling-related mechanisms, initial intervention still remains a challenge. More specifically, they lack active intervention capability in the early stage of injuries, in which immediate hemostasis and early-stage intervention of oxidative stress play key roles. Therefore, real sequence-integrated therapy systems with initial hemostasis are yet to be fully realized. There are also ongoing issues regarding the optimization of the biointerface and stability of the piezoelectric response.

To overcome these challenges, Hu *et al*. 117 developed an integrated piezoelectric hydrogel system that was responsive to ultrasound stimulation and contained Ce-containing carbon dots (CeCDs) **(Figure 3A-B)**. They entrapped dopamine-functionalized barium titanate nanoparticles (PBT) and CeCDs into a network of poly(vinyl alcohol) and sodium alginate via a freezing-thawing process. The objective was to promote the various stages of wound healing through a concerted process that includes hemostasis, inflammation regulation and electric stimulus-assisted regeneration, instead of a single therapeutic effect.

A CeCDs composite was demonstrated to be a highly porous (85 ± 3%) with a well-interim microstructure **(Figure 3C)** containing homogeneously distributed pores with an average pore size of 1.25 ± 0.25 μm. EDS analyses showed Ce and Ti elements were homogeneously dispersed in the hydrogel, where CeCDs and PBT were integrated. **(Figure 3D)** demonstrated the open-circuit voltage response to ultrasound excitation, with the CeCDs composite exhibiting the highest and most consistent response. This improvement came from a combined interfacial polarization and synergistic effect of PBT and CeCDs, performing better than the pure hydrogel.

A strong piezoelectric response was observed in the PVA/SA/PBT/CeCDs hydrogel as well, with its amplitude-voltage curve having a butterfly-like structure in **(Figure 3E)**. Meanwhile, the ferroelectric hysteresis loop in **(Figure 3F)** had a wide and distinct shape, signifying high polarization and output. The addition of PBT also linked the charges. Appropriate inflammation responses are important for wound repair. According to the flow cytometry in **(Figure 3G)**, when CeCDs hydrogel was activated by ultrasound, the number of M2 marker (CD206⁺) positive macrophages increased and number of M1 marker (CD86⁺) positive macrophages decreased compared with the control group, suggesting that the macrophages were in an anti-inflammatory state.

Taken together, these findings demonstrate that the ultrasound-triggered CeCDs hydrogel facilitates wound regeneration by the combined action of biochemical and electrical cues **(Figure 3H).** In this context, CeCDs play a role in antioxidant and immunomodulatory effects and the piezoelectric element regulates bioelectrical signals. This collectively contributes to a pro-regenerative microenvironment with improved vascularization, lower inflammation and collagen organization. The Masson trichrome staining also indicated enhanced collagen regeneration and tissue organisation **(Figure 3I)**.

PZDT is now recognized as an effective wound healing agent, it is notable for its potential to change the treatment of severe periodontal disease. Large dental pockets have long posed a problem for root planning and conventional scaling, as these tools are unable to access each recess. PZDT solves this limitation by using ultrasonic technology, which can penetrate these typically "silent" zones non-invasively and have a dual effect: quick, targeted bacterial killing and immune control. It operates by generating ROS to break down refractory biofilm while concurrently steering the local environment toward bone formation, as demonstrated by increased activity of markers like alkaline phosphatase (ALP) and RUNX2, making the approach really bioactive rather than simply mechanistic. For example, if injectable piezoelectric hydrogels or nanozyme-mediated synergistic devices become conventional therapies, they have the ability to drastically change how alveolar bone healing is approached, moving care beyond basic infection management and into predictable morphological restoration. The combination of strong infection management and energy-activated tissue repair makes piezodynamic therapy one of the more attractive new approaches in dental care.

## 6. Future perspectives

The advancement of piezodynamic therapy from laboratory studies to regular periodontal practice is heavily reliant on the emergence of more complicated, multimodal delivery methods. Integration of piezoelectric components into bioadhesive hydrogels, Janus membranes, and smart implantable devices promises to be a critical area for future development. Such systems may offer prolonged, localized therapeutic responses that include but are not limited to maximized bacterial killing and rapid bone formation in response to physiological cues in the oral cavity. But perhaps the greatest paradigm shift is in *in vivo* energy harvesting where the native stimuli of the stomatognathic system (eating, drinking and swallowing) are harnessed to develop therapeutic devices 119.

Through the use of triboelectric or piezoelectric materials in tooth ceramics or orthodontics, it may soon be possible to produce locally-available micro-currents that can not only eradicate resistant biofilms, but induce cellular bioenergetics to promote wound healing. Moreover, use of these systems could be expanded to the targeted treatment of oral or esophageal cancer through sonodynamic therapy 120. As we innovate with piezoelectric ceramics for restorative dentistry and nano-engineered "injectable or sprayable" formulations, the oral cavity is poised to become a self-regulating, "smart" environment that actively maintains its own health and regenerates lost tissues through mechanical-to-electrical transduction. One factor that gets less attention than it should in ultrasound-responsive periodontal therapy is how variable the periodontal microbiome actually is 121. Periodontitis doesn't stem from a single pathogen, it arises from a dysbiotic microbial community that shifts in composition as the disease progresses 122. As a result, variation in biofilm maturity, microbial diversity, and how much of the keystone pathogens are present could meaningfully shape how well any given therapy performs. In this scenario, microbiome characterization might be a useful tool for directing the sensible choice of functional materials and ultrasonic operating conditions 123. To accomplish successful bacterial eradication in periodontal lesions characterized by dense, mature biofilms, extremely efficient ROS-generating technologies may be required, such as sonodynamic or piezodynamic platforms triggered under cavitation-favorable ultrasonic conditions.

Conversely, following microbial control, lower-intensity ultrasound stimulation combined with piezoelectric biomaterials may be more suitable for promoting periodontal ligament regeneration and alveolar bone repair 124. Therefore, integrating microbiome profiling with material engineering and ultrasound optimization may represent a promising strategy for developing personalized and stage-specific periodontal therapies in the future.

### 6.1 Piezoelectric biomaterials

Biomaterials were first proposed in 1970 by Joseph W. Lechter. Lechter defined biomaterials as natural or synthetic materials intended to react with biological systems and assist in clinical research, practice and development. An explosion of advancement in materials science and the advent of nanotechnology have led to new opportunities for biomaterials research and biomedical advancement 125. One of these is a special group of biomaterials - piezoelectric biomaterials - that can transform mechanical and electrical energy without an external power source. They include materials such as PZT, ZnO, hydroxyapatite (HA), PLLA, PVDF, P(VDF-TrFE) and boron nitride (BN), to name a few 126. They are commonly applied in biomedical applications including sensing, actuation, energy harvesting, catalysis and drug delivery devices. Piezoelectric materials with strong electromechanical coupling such as PZT, PVDF-based polymers, lithium niobate (LiNbO_3_) and bismuth titanate (BiT) are widely known piezoelectric materials. Moreover, two-dimensional materials such as MXenes and graphene are of interest because of their high specific surface area, good electrical conductivity, and biocompatibility 127.

The ability of piezoelectric biomaterials to naturally produce electrical output under mechanical strain is helpful for tissue regeneration and wireless stimulation applications (particularly in dental and oral applications) 128. The clinical use of such platforms is promising due to their self-powered stimulatory effect from natural body movements, low cytotoxicity, few side effects, and good spatial-temporal controllability. Electrical stimuli involving piezoelectric biomaterials can modulate the immune response by transforming the polarization of macrophages from pro-inflammatory (M1) to anti-inflammatory (M2) ones, removing excess foreign body reactions and lowering immunological responses. This might lessen harm and promote recovery. However, these materials must be altered to increase their stability, mechanical strength, and repeatability 129.

A key focus in periodontal treatment continues to be plaque management, and this has led to the emergence of new biomaterials and bioengineering approaches. Piezoelectric materials, for example, are useful due to enhanced efficacy and reduced side effects. Montoya *et al*. 130 studied the interactions of subgingival bacteria with biomaterial systems associated with periodontitis. They discovered that antibacterial BaTiO_3_-based piezoelectric composites, under cyclic mechanical stimulation, produced electrical charges that affected the bacteria's ability to survive. Crucially, this did not notably change the composition of the whole microbiome, and hence prevented microbial dysbiosis. The term non-surgical periodontal treatment (NSPT) was introduced in the 1980s. Over the years, we have learned that infection and its by-products are the main cause of periodontal disease. By understanding the causes and risk factors of periodontal disease, there has been the addition of new technologies to destroy the pathogens and halt the progression of disease.

Roldan *et al.* 131 dsigned an injectable piezoelectric hydrogel (PiezoGEL) system of 200 mg/mL gelatin methacryloyl (GelMA) and 9 mg/mL silanized BaTiO_3_ particles. Liu *et al*. 132 also developed a piezoelectric system made of tetragonal BaTiO_3_ nanoparticles (t-BTO NPs) and tilapia fish gelatin. This system produces electrical stimulation when subjected to external mechanical stimuli, which has effects on immunoregulation and osteogenesis, contributing to periodontal regeneration. Piezoelectric antibacterial biomaterials have a great potential for wound healing and tissue regeneration. But the dependence of the antibacterial effect on electric stimulation strength is not well understood. The challenge to balance the antibacterial and tissue regeneration effects is still ongoing.

Song *et al*. 133 recently explored flexible electroactive BaTiO_3_/P(VDF-TrFE) nanocomposite membranes (EMs) with adjustable surface charge characteristics for infected wound therapy. This team developed these membranes via a solution-casting method by blending dopamine-grafted BaTiO_3_ nanoparticles with P(VDF-TrFE) **(Figure 4A-B)**. Annealing treatments further improved the membranes' piezoelectric properties, such as their surface potential, remnant polarization, piezoelectric coefficient (d33) and output voltage **(Figure 4C-D)**. Moreover, the membranes retained their electrical properties even after a long exposure to culture media, ensuring their *in vivo* reliability. With their high electroactive properties, these EMs also have good antibacterial performance. *In vitro* anti-bacterial tests using Staphylococcus aureus (Gram-positive) and Porphyromonas gingivalis (Gram-negative) bacteria showed that as the electrical potential of the surface increased, the number of colony-forming units decreased, as measured by the dilution and plating method **(Figure 4E-F)**.

For *in vivo* studies, the EMs were used in a ligature-induced periodontitis model, to be challenged by P. gingivalis. Ligatures were removed at day 10 after induction and EMs were implanted as guided tissue regeneration (GTR) membranes into alveolar bone defects **(Figure 4G)**. Micro CT scans at 4- and 8-weeks post-treatment revealed decreased cementoenamel junction-alveolar bone crest (CEJ-ABC) distances and increased bone mineral density (BMD) in the treated group, but no significant improvement in the control groups **(Figure 4H)**.

H&E staining also indicated reduced inflammation and repair of periodontal tissues **(Figure 4I)**. In comparison with the control groups, the high-charge EM group (L3 samples) exhibited decreased inflammation and bone resorption. Fluorescence *in situ* hybridization (FISH) demonstrated significantly low P. gingivalis colonization in the treated group's gingiva tissues, further indicating the antibacterial effect **(Figure 4J)**. Moreover, quantitative PCR and immunofluorescence analyses revealed reduced expression of inflammatory cytokines such as IL-6, IL-1β and IFN-γ in high-charge treated tissues.

In summary, the current study showed a dose-dependent antibacterial and wound-healing effect of the tunable BaTiO_3_/P(VDF-TrFE) electroactive nanocomposite membrane. The system exhibited charge-dependent down-regulation of bacterial viability, both for S. aureus and P. gingivalis. The surface electrical potential increased ROS treatment and prevented bacterial transport across cell membranes to inactivate bacteria. Biocompatibility analysis also showed no cytotoxicity, suggesting it may be safe for implantable applications.

The discussion of piezoelectric biomaterials emphasizes a fast increasing and promising avenue for periodontitis treatment; yet, it is mostly descriptive and lacks the critical synthesis required of a high-level viewpoint. Although the biomaterials structure offers a useful historical viewpoint and the summary of systems, such as BaTiO_3_-based composites, PZDT, and PVDF, is instructive, the argument does not establish whether piezoelectricity offers a clear mechanistic advantage over current electroactive or antimicrobial systems. While basic causative processes, dose-response relationships, and *in vivo* reproducibility are mainly ignored, the story presents electrical stimulation as automatically inducing immunomodulation and osteogenesis. Research on BaTiO_3_-based systems shows improvement in bone-related metrics and antibacterial efficacy, but the absence of any measurable change in microbiome composition begs the issue of whether these materials are actually curing dysbiosis or are only temporarily reducing bacterial load.

Similar challenges arise when claims about immune regulation and macrophage polarization are put forward without accounting for the possibility of variations in periodontal microenvironments, stimulation settings, or material composition. Electrical heterogeneity in *in vivo* contexts is one significant gap; unequal or poorly regulated charge generation may lead to varying therapeutic effects, which are not adequately addressed. Additionally, the study prioritizes material innovation above practical translational preparation, giving little attention to long-term stability, manufacturability, and compatibility with proven non-surgical periodontal therapies. Clinically meaningful norms, mechanistic validation, and standardized electroactive response measurements would take precedence over just listing items in a more rigorous method.

#### 6.1.1 Ultrasound responsive hydrogels

Owing in large part to their excellent biocompatibility, adaptable mechanical properties, and capacity to conform the complex architecture of the mouth cavity, ultrasound-reactive hydrogels represent a promising class of multifunctional platforms for targeted, controlled dental therapy 134. In order to enable on-demand activation by ultrasonic stimulation for greater ROS generation and targeted biofilm clearance, these devices can incorporate sonosensitizers, antibacterial compounds, or piezoelectric components. Additionally, its hydrated network allows for longer drug release and better retention at the medication site, both of which are highly beneficial in dynamic oral conditions.

Hydrogel-based implants are extremely appealing for applications such as periodontitis, peri-implantitis, and wound recovery because they combine responsiveness to ultrasound and local stimuli (e.g., pH, enzymes) to increase antibacterial activity while also stimulating tissue repair 135-137. Chen *et al*. 138 constructed a supramolecular nanoreactor that combines photothermal conversion, cascade reactions, and controlled hydrogel formation. It successfully destroys bacterial biofilms and accelerates healing of tooth extraction wounds by establishing a localized and sustained treatment environment.

Solanki *et al*. 139 developed several multifunctional and bioresponsive piezoelectric dental systems, such as dental restorations with carcinogenic-antimicrobial-remineralization activity, prosthodontic dentures with antifungal activity, and piezoelectric hydrogels for antibacterial and bone regeneration in the periodontal region. These multi-responsive materials showed that mechanically derived electricity can eliminate biofilm, boost osteogenic differentiation (cell bone formation) and deposit mineral.

Zhou *et al*. 33 developed double-salt strategy in a polyvinyl alcohol (PVA) hydrogel using MgCl_2_ and Na_3_Ct. The system enhances ionic conductivity and the polymer structure through multivalent coordination and ionic regulation. The hydrogel is injectable, mouldable to periodontal defects and facilitates charge carriage. Importantly, Mg^+2^ plays a dual biological role by guiding macrophage polarization and activating osteogenic signaling pathways, thereby supporting both immune regulation and bone regeneration. This multi-component hydrogel amplifies the piezoelectric response and enhances mechanical, electrical and injectability properties of the hydrogel. In summary, it provides precise control of mechanical, electrical and immune cues in the periodontitis environment to allow simultaneous and coordinated bone regeneration and immune remodeling to repair the periodontium.

Wang *et al*. **140** designed a supramolecular nucleoside hydrogel via self-assembly of chemically modified PEGylated nucleosides. It is based on a supramolecular network via multivalent hydrogen-bonding interactions. This hydrogel has a high therapeutic potential for periodontitis using a cascade therapy that combines and enhances antimicrobial and anti-osteoclast activity. Guided bone regeneration (GBR) is required for more than 60% of patients restored with dental implants to yield adequate bone volume. Yet, poor and late gingival closure has considerably negative effects on bone regeneration for several reasons. First, bacterial infection is a serious concern because about 9.25%-19.5% of implants are affected by peri-implantitis and the incidence is higher when the gingiva is not properly sealed. Second, antibiotics pose issues of drug resistance and cytotoxicity by metal nanoparticles that limit their use. Second, bone graft material loss is an issue as long-term stability of the graft material is crucial for GBR, but its loss occurs due to insufficient soft tissue coverage, resulting in degradation or loss. Third, fibrous tissue infiltration interferes with bone regeneration and increases surgical complexity, especially when barrier membranes are used. While autologous gingival flap (AGF) grafts can be used to reinstate soft tissue coverage, they cause a second injury and may destabilize the flaps.

Consequently, new methods to provide antimicrobial protection, differentiation barrier and simultaneous gingival and bony regeneration for patients with diminished bone and soft tissue volume are urgently desired 141-143. Zhu *et al*. 144 used a biomimetic strategy for gingival tissue engineering to overcome these challenges. Gingiva is a three-layered functional structure, including an external epithelial layer to afford antibacterial protection and facilitate tissue attachment in part through epithelial pegs, a middle lamina propria that allows for structural support and nutrient diffusion (mainly consisting of fibroblasts and collagen) and an inner periosteal layer to prevent the ingrowth of fibrous tissue and to participate in the osteogenic process. The hierarchical structure led to the design of a multilayer biomimetic gingival flap (BGF) **(Figure 5A)**. The ¹H nuclear magnetic resonance (NMR) and Fourier transform infrared (FTIR) spectra confirmed the reaction between Gel-L and PLMA. Gel-EPL showed a dramatic decrease in signals corresponding to vinyl protons (5.77 and 6.18 ppm), methyl group next to the double bond (1.96 ppm) and methylene within the amino ortho position (3.26 ppm) compared to non-crosslinked PLMA **(Figure 5B)**. FTIR spectra also confirmed this reaction, with a reduced intensity at 3080 cm^-1^ (C=C stretching) and 3282 cm^-1^ (N–H stretching) in Gel-EPL compared with PLMA, indicating successful chemical reaction and crosslinking. Gel-EPL exhibited a lower peak intensity in the 1720 cm^-1^ region (C=O stretching) and 1500-1700 cm^-1^ region (amide I, II and III bands) compared with Gel-L, suggesting structure modification by crosslinking.

Microscopic images showed obvious phase separation and emulsified particles in Gel-PEO **(Figure 5C)**, but no phase separation in Gel-L. Higher PEO concentrations resulted in emulsified droplets with three different size distributions. PEO release profiles showed that it released quickly (within 3 hours), before slowing down over 24 hours and finally leveling off. Antibacterial activity is essential in BGF, particularly as peri implant materials are easily infected by oral microbes. The effects of Gel-EPL on Staphylococcus aureus, Porphyromonas gingivalis and Streptococcus mutans indicated that the system inhibited growth of all three strains, when assessed by colony counting **(Figure 5D)**. This was supported by histological assessment of tissue regeneration. A hematoxylin and eosin (H&E) staining indicated that WE and GBR groups both showed incomplete regeneration of both the epithelial and lamina propria layers, though it was noted that the GBR group showed severe inflammatory cell infiltration around the graft area. But AGF and BGF groups resulted in complete healing and formation of epithelial pegs, with an unstable interface between the epithelial and lamina propria layers in AGF group **(Figure 5E)**. Masson’s trichrome and Sirius Red staining showed that AGF and BGF groups had a greater presence of collagen (mainly type I) with a more dense and parallel arrangement compared to other groups.

Despite the fact that ultrasound-activated hydrogels are described as adaptable frameworks for localised dental treatment, the discussion is mostly descriptive and fails to sufficiently address real limitations or the challenges of putting this work into clinical practice. Different hydrogel methods must be methodically examined in terms of stability, the depth to which ultrasonic penetrates oral tissue, and performance under clinically relevant circumstances in order to support the claims of enhanced ROS production, biofilm destruction, and tissue regeneration. As of right now, the revealed complex multifunctional system reads more like a singular instance than a noteworthy illustration of the broader hydrogel therapy field.

The sections on guided bone regeneration and biomimetic gingival flap formation are helpful on their own, but they appear detached from the main problem of ultrasonic responsiveness, which weakens the argument's overall coherence. Important limitations including degrading behaviour, long-term biocompatibility, and control over drug-release kinetics are not taken into account, nor is there a molecular explanation of how ultrasonic specifically impacts hydrogel performance. The importance of this section might be greatly increased by a more thorough explanation connecting material design, ultrasonic activation, and clinical implementation.

#### 6.1.2 Janus membranes

Due to its asymmetric shape and two physiologically distinct surfaces, Janus membranes have become a promising approach to improve dental treatment, especially guided tissue repair 143. Several, frequently incompatible functionalities can live on one platform because to this structural mismatch. For instance, one face can encourage tissue integration, osteogenesis, and cell adhesion while the other face acts as a barrier against fibrous tissue infiltration and bacterial invasion 145. Janus membranes acquire an additional layer of spatiotemporal control when paired with ultrasound-responsive components, enabling greater regeneration signaling and on-demand ROS production for antibiofilm actions 146.

These membranes are especially well suited to periodontitis and peri-implantitis, which need coordinated regeneration of both soft and hard tissue, since they may mimic the layered architecture of natural dental tissue 147. Collagen-based GBR membranes can be greatly enhanced by biomimetic mineralization of collagen. Natural bone is a complex composite material consisting of an organic collagenous matrix and an inorganic mineral phase, mostly hydroxyapatite (HAp). This complex assembly is arranged hierarchically, from the microscopic configuration of collagen fibrils to the macroscopic bone forms 148. The two main mechanisms that control the spatial distribution of HAp with regard to collagen fibrils in biomineralization a crucial step in the production of bones are intrafibrillar and extrafibrillar mineralization. Because it explains the exact deposition of HAp nanocrystals within the empty spaces of collagen fibrils, which is necessary for the remarkable mechanical capabilities of original bone, the intrafibrillar technique is very convincing. To further understand and reproduce this appealing natural phenomenon *in vitro*, researchers created the polymer-induced liquid precursor (PILP) idea.

This theory states that amorphous calcium phosphate (ACP) precursors are stabilized by polyelectrolytes such polyaspartic acid (PAsp), poly(acrylic acid) acid (PAA), and poly(allylamine hydrochloride) (PAH). These stabilized precursors are able to pass via the D-periodic gap zones, which are naturally occurring gaps that appear along collagen fibrils at periodic intervals 149.

These amorphous phases undergo controlled crystallization after infiltration, producing well-ordered hydroxyapatite nanocrystals that are confined inside the fibril. When used in bone tissue engineering, collagen constructs mineralized in this way where crystals develop inside the fibrils rather than just outside them have shown clear advantages over pure collagen scaffolds or those mineralized only on the outside. They are a more biomimetic scaffold overall because of their strong resemblance to the nanostructure of real bone, which leads to improved mechanical efficiency and a far greater potential to induce regeneration 150.

Research has increasingly concentrated on sophisticated biomimetic techniques for optimising the composition and structure of collagen-based biomaterials, building on this basic understanding of intrafibrillar mineralization. Research in this area has shown that *in vitro* mineralization techniques are flexible enough to include inorganic phases other than hydroxyapatite into collagen fibrils. Alternatives including silica, yttria-stabilized zirconia, calcium carbonate, and calcium fluoride each offer special qualities and potential medical uses depending on the application 151.

Among these, strontium stands out due to its wide-ranging effects on bone metabolism, which have caught the attention of researchers. By promoting osteogenesis, or the growth of new bone, and preventing bone resorption, or the breakdown of existing bone, strontium supports a healthy balance in bone remodeling.

Strontium-functionalized nanomaterials for skeletal healing have drawn more attention due to their dual osteoanabolic and anti-catabolic properties. Intrafibrillar mineralization of collagen with strontium apatite has been demonstrated by recent proof-of-concept studies, suggesting a feasible route for directly integrating this advantageous component into scaffolds that imitate bone 152. These encouraging results have led scientists to suggest that a collagen intrafibrillarly mineralized with strontium apatite guided bone regeneration (GBR) membrane might greatly improve bone-healing results. Although this concept offers enormous promise for creating regenerative techniques, much more study is needed to fully understand its clinical and mechanistic implications 153. Despite the significant evolution of collagen-based GBR membranes, one enduring drawback is their insufficient barrier function against undesired soft tissue infiltration. This weakness stems from the naturally fibrous and porous nature of collagen, which continues even when mineral phases are added 154. In order to prevent fibrous connective tissue from growing into the bone defect, a thick, robust protective layer should be applied to one face of the membrane. For this, Janus materials named for the two-faced Roman god and distinguished by their asymmetrical structure offer the perfect design template. The ability to carry different chemical, physical, or biological characteristics on either face, enabling directed function, is what sets them apart. Janus systems have become more attractive in biomedical applications because to their anisotropic nature, especially when biological responses need to be spatially managed, such guiding tissue regeneration 155. The layered requirements of guided bone regeneration are especially well-suited to Janus membranes since each surface may be independently designed for a unique, overlapping function.

For example, one of them can be built to physically stop soft tissue invasion, while the other promotes and accelerates the growth of bone. For the bioactive, porous element of this two-part system, intrafibrillarly mineralised collagen injected with strontium apatite is a potential option 156. This material's bone-mimicking nanostructure and intrinsic osteoinductive properties, which are brought about by strontium ion release, provide strong support for osteogenesis. Additionally, it has been shown to promote angiogenesis the growth of new blood vessels necessary for tissue survival as well as immunomodulatory effects that might promote a more favourable healing environment 157. In contrast, the thick barrier layer needs completely different characteristics, such as simplicity of manufacture, predictable degrading behaviour, and long-term mechanical durability.

In the field of biomedicine, aliphatic polyesters a type of synthetic biodegradable polymer valued for its biocompatibility and adjustable properties are widely used substitutes. Polycaprolactone (PCL) is especially well-known for its use in bone regeneration applications. Its great mechanical stability and low rate of degradation help maintain structural space inside a defect over time, which is necessary for successful bone ingrowth 158. Polycaprolactone methacryloyl (PCLMA) is created by chemically modifying PCL with methacrylate groups, which enhances its processability and industrial adaptability 159. By adding photocurable groups throughout the polymer backbone, this modification enables the modified polymer to rapidly photocrosslink when exposed to blue light in the presence of a sufficient photoinitiator. PCLMA is instantaneously changed from a viscous liquid to a solid, mechanically robust scaffold by this light-activated polymerization. This characteristic makes PCLMA perfect for creating a Janus membrane's thick, load-bearing outer layer, enabling exact form and integration control 160,161.

By developing a multifunctional GBR membrane with well-optimized properties, Zhao *et al*. 162 demonstrated this Janus design method. An inner layer of collagen that would be in close proximity to the bone defect and intrafibrillarly mineralised with strontium apatite was part of their concept. Precursors of amorphous strontium phosphate stabilised with poly(acrylic acid) (PAA-ASP) enabled precisely controlled mineralisation, guaranteeing the mineral was deposited straight into the collagen fibrils. Both a support structure and a physical barrier were provided by the outer layer, which was composed of enlarged PCLMA to improve the bioactive core **(Figure 6A)**. Adding PCLMA while it was still liquid and uncured allowed it to progressively seep into the underlying collagen matrix's interfibrillar spaces, which was a crucial component of their building method. Microscopy revealed a highly linked, nano-interlocked interface between the two layers as a result of photo-crosslinking of the PCLMA brought on by blue light irradiation. The researchers used molecular dynamics (MD) simulations on the assembly of PAA-ASP and PAA-ACP systems to gain a better understanding of the processes behind precursor formation **(Figure 6B)**. The simulations were performed in a TIP3P water environment, which allowed for close monitoring of molecular dynamics.

PAA polyelectrolyte was seen in experiments as cyan chains, HPO_4_^2^⁻ ions as red clusters, Ca^2^⁺ ions as yellow spheres, and Sr^2⁺^ ions as grey spheres. A considerable number of Sr^2⁺^ and HPO_4_^2^⁻ ions gathered around the PAA chains by 80 nanoseconds into the simulation, creating stable PAA-ASP clusters. This sheds light on how the polyelectrolyte controls the stabilization of mineral precursors. The chemical makeup of the membranes was verified using Fourier transform infrared (FTIR) spectroscopy **(Figure 6C-D).** The findings demonstrated the structural integrity of PCLMA thru clear and distinct absorption peaks: C-O-C vibrations at 1160 cm^-1^, a strong C=O stretching vibration at 1731 cm^-1^ revealing the ester groups, and symmetrical and asymmetrical -CH2 stretching vibrations at 2941 and 2861 cm^-1^. All three examined membrane variations the strontium-modified Janus membrane (SrJM), the calcium-functionalized variant (CaJM), and the non-mineralized control (JM), consistently exhibited amide I and amide II bands. Despite the several processing steps, these bands spectroscopic markers of peptide connections typically observed between 1635 cm^-1^ and 1550 cm^-1^ showed that collagen was still present inside the composite nanostructures.

The specific mineral phase present in the strontium-rich membrane (SrJM) was identified owing to further structural information obtained from X-ray diffraction **(Figure 6E)**. Numerous distinct diffraction peaks that closely matched the crystallographic planes of strontium apatite were visible in the XRD patterns. Strontium apatite has successfully crystallised as the primary inorganic phase inside the collagen matrix, according to reflections matching to the (211), (300), (310), and (222) planes. The mineral and organic content of each membrane variant was subsequently measured by thermogravimetric analysis (TGA) **(Figure 6F)**. Due to the removal of physically adsorbed water, the thermographs consistently showed an early mass loss below 150°C with a clear derivative peak about 50.8°C. A clear derivative peak at 324.5°C shows that mass loss rose dramatically over 150°C, showing thermal breakdown of the organic components, including the PCLMA polymer and the collagen matrix. The material's specific composition was determined by the thermal analysis. Histological examination and advanced imaging were used to assess the produced membranes' capacity for regeneration. Cellular morphology and collagen deposition patterns in the defect regions were shown by Masson's trichrome staining and haematoxylin and eosin (H&E), demonstrating the formation of new bone **(Figure 6G)**.

Extensive three-dimensional analysis was made possible by micro-computed tomography (micro-CT) modelling, which showed that the SrJM group had much more bone regeneration than the other membrane types **(Figure 6H)**. After a 12-week implantation period, the defects treated with the SrJM demonstrated nearly complete bone repair, robust tissue integration, and structural integrity. The limited regeneration of CaJM group and the non-mineralized JM and lack of recovery demonstrate the unique beneficial role of strontium and the intrafibrillar mineralization technique. Quantitative analysis of the micro-CT data provided substantial statistical support for these qualitative results.

Important bone morphometric measurements were significantly higher in the SrJM group, including bone mineral density (BMD) (388.62 mg HA/cm^3^), bone volume fraction (BV/TV) (72.19%), and bone surface density (BS/TV) (10.01 mm-1) **(Figure 6I)**. Together, these quantitative criteria showed that the SrJM membrane produced more and higher-quality new bone.

In conclusion, a significant advancement in GBR approaches is the sophisticated SrJM Janus membrane, which meticulously blends an intrafibrillarly strontium-apatite-mineralized collagen layer as the primary bioactive phase with a robust, photo-crosslinked PCLMA barrier layer. The precise control over mineral deposition is attributed to the PAA-ASP system, which effectively mediates the controlled intrafibrillar mineralisation process by directing the deposition of strontium apatite nanocrystals within the collagen fibrils.

Concurrently, the PCLMA layer is intended to reinforce the overall structural integrity of the membrane. The resulting reinforcement creates a nanointerlocked interface that guarantees strong adhesion and mechanical continuity between the layers thru rapid photo-crosslinking. When considered collectively, this meticulously crafted composite structure provides several noteworthy advantages, such as robust mechanical stability that promotes space preservation and resistance to external forces; a degradation profile modified to allow the membrane to endure long enough to support bone regeneration while being gradually resorbed; and, perhaps most importantly, sustained strontium ion release that provides ongoing therapeutic support for osteogenesis and bone homeostasis. When integrated, these features allow the SrJM Janus membrane to meet the essential requirements of an effective guided bone regeneration device, indicating a workable path toward better bone-healing outcomes in clinical use.

Although the idea of combining intrafibrillarly activated collagen with a photo-crosslinkable PCLMA layer is intriguing, it is unclear what specific advantages this asymmetric Janus design offers over current GBR membranes, particularly in terms of how each layer affects tissue integration, osteogenesis, and barrier performance. The membrane is described as a sophisticated, multifunctional system, but rather than critically analyzing its practical utility, the discussion concentrates on physicochemical characterization. Fundamental translational issues including scalability, long-term degradation behaviour, and whether *in situ* photo-crosslinking is actually feasible in the complex oral environment remain unanswered despite noted advancements in bone regeneration. It is also uncertain how beneficial strontium synthesis is in comparison to other bioactive systems. In general, this section could use a more balanced, integrated approach that links clinical application to material design and places the system in the larger context of GBR technology.

##### 6.1.2.1 Piezoelectric Janus Membranes

In contrast to materials used for cancer therapy treatment or skin wound care, periodontal materials must be tailored for the dynamic environment of the oral cavity, where saliva, enzymatic degradation, microbial re-colonization, and chewing forces can compromise material retention and/or therapeutic effectiveness. Coordinated regeneration of the periodontal ligament, cementum and alveolar bone is also required for periodontal regeneration, in addition to infection and inflammation control.

Therefore, ultrasound-responsive periodontal materials should be engineered with appropriate cohesion, tissue retention, mechanical stability, controlled stimulus responsiveness, and biocompatibility for effective intraoral application. Many efforts have been made to develop GTR membranes containing antibacterial agents. A number of studies have involved osteogenic enhancers to enhance osteogenesis of these membranes 163. However, a major drawback of these strategies is the challenging ability to control the delivery of drugs. This leads to acute release and localized accumulation of these chemicals, which causes toxicity and biocompatibility issues. It's generally known that positively charged species have strong anti-bacterial properties, as bacterial membranes are highly saturated with negatively charged species, which can be disrupted by the repulsive interaction. On the other hand, such surfaces can facilitate the binding of positive ions, e.g. Ca^2+,^ in solution, thereby promoting biomineralization, and facilitating Ca^2+^ uptake into cells and promote osteogenic activity. These two characteristics, one positively and the other negatively charged, may be exploited in combination in a Janus charge based GTR membrane. Such an unequal design may simultaneously enhance the antibacterial and osteogenic properties, thereby eliminating bone resorption caused by periodontitis but avoiding complications resulting from controlled release of drugs. Teeth in the oral environment are subject to continuous biting forces and therefore under these mechanical forces in the oral cavity piezoelectric materials may generate electrical outputs of significant magnitudes, making them an ideal choice to elect a Janus membrane GTR for treatment of periodontitis. Application of electric charges or cyclic compression forces to piezoelectric materials aligns dipoles, making surfaces with different polarities (positive and negative) 164-166.

This advantage allows repolarizing the piezoelectric Janus membrane and its subsequent placement in the periodontal window with the positively charged side towards the gingival tissue to eradicate bacteria and the negatively charged side towards the bone defect to promote osteogenesis 167. One particular feature of piezoelectric materials is their capacity to generate electrical impulses autonomously, removing the need for external power sources and giving continuous electrical stimulation to cells, so promoting tissue regeneration. Membranes made of piezoelectric materials are very appealing for periodontal regeneration because they may send electrical signals to cells. Mechanical forces generated during mastication function as a constant stimulus, activating and intensifying the piezoelectric response, resulting in increased electrical output. This electrically active milieu not only directly effects bacteria and bone-related cells, but it also plays an important role in immunomodulation by encouraging the development of anti-inflammatory M2 phenotype macrophages. Creating the optimal environment for alveolar bone repair 168. Currently, there are a few researches on applied piezoelectric composites including barium titanate nanoparticles for periodontal restoration, but they do not incorporate an asymmetric charge distribution to supply the membrane with activity to combat bacteria 169,170.

Using electrospinning technique, Li *et al*. 171 designed a biodegradable piezoelectric Janus membrane made up of two asymmetric fibre layers. The outer antibacterial layer, as shown in Scheme 1b, is made of electrospun pure PLLA (P) membrane, which has potent antibacterial activity, while the inner osteoinductive layer is made of electrospun PLLA/gelatin (PG) composite membrane, which has high hydrophilicity and cell/tissue compatibility, allowing for easy implantation **(Figure 7A)**. Before being merged, the P membrane is annealed to produce an A-P membrane, which is then joined to the PG fibre.

The morphology of the membranes was examined using SEM, which revealed the nonwoven fibre arrangement for the P, A-P, and PG membranes. The average diameters of P, A-P, and PG fibres were 0.59 ± 0.20, 0.85 ± 0.24, and 0.86 ± 0.21 μm, respectively. **Figure 7B** depicts the cross-sectional image of the Janus membrane A-P/PG, with the two layers densely packed together. The fibroblasts grew effectively on all substrates but entered the porous structure of the collagen foam, while remaining mostly on the surfaces of the A-P and Bio-Gide membranes. Given that the Bio-Gide membrane is commonly employed as a GTR barrier in clinical applications, the A-P membrane's ability to successfully limit fibroblast penetration validates its eligibility for GTR treatment in contact with gingival tissues. Given that the Bio-Gide membrane is commonly employed as a GTR barrier in clinical applications, the A-P membrane's ability to successfully limit fibroblast penetration validates its eligibility for GTR treatment in contact with gingival tissues. The mechanical characteristics of the P and A-P membranes were also evaluated, as seen in the inset of **(Figure 7C)**.

The A-P membrane had somewhat higher tensile strength than the P membrane, but it had a significantly lower elongation at break, reducing from roughly 175% (P) to 82% (A-P). This drop-in elasticity is most likely caused by annealing-induced changes in the crystalline structure of PLLA inside the fibres. In **(Figure 7D)** the C-V curves revealed that the A-P membrane had a wider hysteresis loop than the P membrane, showing that the annealing treatment improved the electrochemical properties of PLLA membranes. Notably, the A-P (+) membrane had the biggest C-V hysteresis loop of any sample, the electrochemical property of the P (+) was stronger than the P, and both results validated the role of polarization. Most persuasively, the piezoelectric effect of the membranes in response to compression was measured with an electrometer, and the results were consistent with the previous findings **(Figure 7E)**. The output voltage of the A-P membrane was measured at different applied pressures (5, 10, and 15 N), indicating a positive association between applied pressure and output charges. The A-P membrane produced a consistent electric output of 0.8 mV during cyclic compression, which was much higher than the P membrane's output voltage of <0.2 mV **(Figure 7F)**. Taken together, the foregoing results showed that the A-P fibres were highly electroactive, with rich charges on the surface following polarization or cyclic compression, ensuring their usage as the outer layer of the Janus membrane to face the gingival tissue for antibacterial properties. As previously stated, the inner side (PG) of the Janus membrane contacts the alveolar bone defect, which was made of PLLA and gelatin at a weight ratio of 1:1. Considering the piezoelectricity of the PLLA, the electrochemical properties of the PG fibres was evaluated. The mouse periodontitis model was established using the ligation approach. A ligature was wrapped around the cervical region of the maxillary second molars on both sides **(Figure 7G)**. To infect the mice, 109 cfu/mL of P. gingivalis was injected daily at the ligature sites for 10 days. The palatal gingiva of the maxillary second molar was raised, and Janus membranes were inserted on its palatal side for therapy. The A-P(+) side confronted the gingival tissue, whereas the PG(-) side faced the alveolar bone. The Sham and Blank groups represented healthy and periodontitis-induced animals without membrane implantation, respectively. In **(Figure 7H)**, the results demonstrated that the expression levels of these three inflammatory genes (IL-1β, IL-6, and INF-γ) in the Blank group were considerably higher than the Sham group. In the Heal-All therapy group, inflammatory cytokine expression was reduced as compared to the Blank group. However, as compared to normal mice without induced periodontitis, the Heal-All group's gingival tissue expressed significant levels of inflammation-related genes, showing that the Heal-All collagen membrane lacks anti-inflammatory characteristics. An aforementioned *in vitro* investigation found that neither layer of the P/PG bilayered membrane exhibited apparent anti-inflammatory properties. Similarly, the gene expression levels of inflammatory cytokines in the gingival tissue of mice treated with the P/PG were similar to those in the Heal-All group, with relatively high expression levels, indicating that treatment with the P/PG membrane does not alleviate periodontal inflammation. Notably, only the A-P(+)/PG(-) membrane has immune-modulating and anti-inflammatory properties. Mice with periodontitis treated with the A-P(+)/PG(-) membrane had significantly lower gene expression levels of all three inflammatory cytokines compared to the blank group.

An intriguing effort to combine osteoinductive and antibacterial properties in an asymmetric bilayer design is the electrospun piezoelectric Janus membrane; unfortunately, the discussion is mostly descriptive and lacks a critical assessment of its wider importance. While the conceptual division of roles between the PLLA outer membrane and the PLLA/gelatin inner membrane makes sense, the reasons why this design is a clear improvement over existing GTR membranes are not clearly defined beyond basic compatibility and antibacterial properties. While the large focus on morphological, mechanical, and electrochemical measurements are informative, they do not adequately tie these traits to clinically relevant properties, particularly in the highly dynamic mouth environment. Additionally, the reported reduction in elasticity following annealing may compromise durability and mechanical strength, which are not discussed. Although the polarized membrane has been shown to have anti-inflammatory benefits in *in vivo* investigations, the underlying mechanisms including how precisely piezoelectric stimulation may affect immune activity remain mostly unknown. Furthermore, there is no discussion of scalability, polarization uniformity, or the durability of electroactive characteristics after implantation. In conclusion, important connections between material creation and function and therapeutic applications would greatly increase the significance of this component.

#### 6.1.3 Piezoelectric ceramics

Ceramics for restorative dentistry must meet many main characteristics, including biocompatibility, aesthetic features, and mechanical performance, to assure the dental equipment' dependability and longevity. Keeping in mind such criteria and relevant applications 172,173.

In recent decades, dental implants have been built using a variety of material species, from ceramic to gold. Nevertheless, the sensory feedback of natural teeth is not fully restored by current implants, which are mainly intended to restore masticatory function. Lack of sensory input affects how food is perceived and makes it more difficult to control chewing force. This flaw might lead to needless overload, which would significantly restrict their clinical outcomes by causing biological failures including temporomandibular joint injury and bone loss as well as technological issues 174,175.

The periodontal ligament (PDL) and alveolar bone that surround the tooth roots are the source of masticatory perception of natural teeth. There are many mechanoreceptors in the PDL, such as Ruffini and Type 3 endings, and periodontal nerves in the alveolar bone that can produce neuroelectric signals to the brain in response to human chewing force 34. These signals would help to stimulate neural activity in the somatosensory cortex for masticatory perception. On the other hand, the inability to provide neuroelectric impulses to the brain is indicated by the disappearance of the PDL around the artificial dental implants. Clinical researchers have worked hard to give dental implants the capacity to respond to force 176,177. Chen *et al.* 35 developed a self-powered dental implant incorporating highly piezoelectric biomaterials that converts normal occlusal forces into bioelectrical signals, providing adaptive multifunctional effects for the early prevention of Peri-implantitis through a rationally engineered piezoelectric lattice design.

Cao *et al*. **178** provided a different approach to restore sensory input using a unique dental crown made of piezoelectric materials, known as a piezoelectric implanted tooth (PIT) **(Figure 8A)**, as opposed to the neuroelectric signals produced by mechanoreceptors inside the periodontal areas. A 3D-printed and assembled implanted tooth with a piezoelectric core and strong sheath was created. This tooth can act as a "mechanoreceptor," translating mechanical chewing force into electrical impulses. The brain may reconstruct the sense feedback loop for masticatory perception by receiving the produced signals via the surrounding nerve system. The viability of a piezoelectric implanted tooth for masticatory perception has been demonstrated by the real-time monitoring of neurological activity in the mouse brain in response to occlusal stimuli.

In addition, compared to patients with existing commercial ceramic dental crowns, more than 90% of patients in clinical instances acknowledged the restoration of their masticatory perception following the implantation of the piezoelectric tooth. This masticatory perception exhibits distinct sensory sensitivity and encompasses a wide spectrum of individuals with various clinical histories. SEM was used to characterize the cross-sectional morphology of the ZrO_2_/BCZT contact. It is evident that there are no visible fissures between the ZrO_2_ and BCZT layers. The presence of Zr, Ca, Ba, and Ti was further confirmed by the multi-element EDS mapping pictures of the zirconia- barium calcium zirconate titanate piezoelectric type (ZrO_2_/BCZT) interface, showing good coalescence of two types of ceramics **(Figure 8B)**. Additionally, following electrical polarization, the piezoelectric characteristics of several PITs (including human and mouse samples) were examined **(Figure 8C-E)**. Both PITs exhibit patterns of rising electrical output with increasing pressures under the induction of various applied pressures. A human tooth's occlusal force can often reach about 250 N. The PIT's output voltage at this pressure is 108 ± 5 mV. Additionally, the PIT's extended lifespan and dependability were evaluated. The findings demonstrate that even after 10,000 cycles of periodic compression and release, its voltage output performance is constant. A unique neuroimaging experimental setup was built in mice to use the PIT to study the natural feedback of occlusal perception **(Figure 8F)**.

Lastly, with the assistance of volunteers in need of dental implants were assembled and an attempt was done to verify the clinical potential of PIT. With their individual agreement, a total of 23 participants who satisfied important inclusion criteria were enlisted for data collection and clinical assessment. In order to get their baseline demographics and illness features, participants who had obtained stable dental implantation and were prepared for phase 2 prosthetic surgery were initially consulted **(Figure 8G)**. There were 11 female volunteers and 12 male participants, with a median age of 44. The experiment was separated into 3 groups: installation of commercial implanted tooth, installation of piezoelectric tooth and a natural tooth on the opposite side of the implanted tooth for biting and chewing test. In addition, PIT demonstrated over 70% comfortable-level intensity (sensation without stimulation or pain) in all four biting studies, demonstrating that its piezoelectric conversion potential is appropriate for occlusal sensing while retaining biocompatibility and bio-comfortability. Finally, a production and assembly method were successfully built to create piezoelectric dental implants. The developed prosthetic tooth enables translation of occlusal force into electrical impulses up to the sensory brain, offering light on a solution to the essential clinical challenge of masticatory perception post tooth implantation.

Although the development of a piezoelectric implanted tooth offers a promising path toward the restoration of masticatory perception, its future translational trajectory is yet unclear and requires closer examination. At the systems neuroscience level, the core hypothesis that mechanically produced electrical impulses may consistently replace natural periodontal mechanotransduction remains unproven, especially when it comes to signal specificity, integration, and long-term cerebral adaptation. To determine if such artificial input may accomplish consistent, high-fidelity sensory restoration across a variety of patient groups, future research must go beyond proof-of-concept demonstrations in small cohorts and short-term animal models. Standardized, quantifiable frameworks for evaluating masticatory perception are equally important since current subjective measurements of "comfort" or "restoration" are insufficient as a foundation for clinical benchmarking. The engineering properties of the initial integrity and piezoelectricity of the interfacial layers are encouraging but the long-term effects of cyclic loading, saliva and enzyme reactions, and pH fluctuations have not been extensively studied. Also, long-term biocompatibility, whether repeated electrical stimulation will be harmful to other neural circuits and tissues in time. The wider availability of this technology in the clinic will also require consideration of issues of production repeatability, cost-effectiveness and regulation. If these issues are not solved, the idea may not make any real dent in the clinical problem, and only be a nice experiment.

### 6.2 Piezodynamic therapy and chemodynamic therapy

The majority of the recent studies focusing on PZDT have been about the generation of ROS as the key mechanism for its therapeutic activity. In this study, the electrical signals that naturally occur within PZT materials when they encounter normal body motion are harnessed as an additional source of ROS and combined with the ROS generated by the PZDT to effectively clear bacteria in infected mouse models 179. This method can be used to manipulate immune function at burn sites for an active anti-infective multimodal therapy. We also combine chemodynamic therapy (CDT) with a PZDT (photoselective destruction of bacteria) within a thermoresponsive hydrogel matrix enabling a precise control of the antibacterial activity in time and space. The outcome is a combination therapy that not only promotes microbial clearance, but also supports tissue repair, so as to complement the regeneration process 180,181.

Piezoelectric biomaterials are a new branch of biomedicine that allows the conversion of mechanical energy derived from normal physiological activities to a therapeutic electrical signal. A more comprehensive paradigm that integrates electromechanical stimulation, immunological regulation, and tissue regeneration is developing, whereas early PZDT investigations have concentrated on the production of ROS for antibacterial and anticancer purposes. The change reflects both the growing understanding of how biological systems respond to mechanical feedback and the potential to use endogenous mechanical signals (body motion, fluid flow, occlusal forces) to create therapies that are more localized, self-powered, and non-invasive. Dentistry comes up as a particularly intriguing but unexplored application sector since the oral environment is continuously subjected to dynamic mechanical stress and is highly susceptible to biofilm-associated infections and inflammatory diseases. The combination of piezoelectric materials with dental science enables the creation of smart implants, coatings, and regenerative scaffolds that may convert masticatory forces into therapeutic electrical or chemical outputs 182,183. However, achieving this goal would necessitate overcoming a number of obstacles, such as long-term safety of continuous stimulation, accurate modulation of electromechanical responses under various stresses, and material stability in the complex oral environment.

To fully realize the promise of piezoelectric-driven treatments in oral healthcare, future research must concentrate on connecting basic materials design with clinically relevant dental applications 184,185. Roy *et al*. 186 introduced a dual-modal device that combines hydrogel matrices with ultrasound-activated piezoelectric and chemodynamic functionalities as a therapeutic approach. They created a technique to increase lattice asymmetry and stabilize piezoelectric domains, both of which enhanced system performance and increased the piezoelectric coefficient. They were able to enhance the polarization effects caused by ultrasonic stimulation and speed up the formation of ROS by combining accurate atomic-level ordering with regulated co-doping of copper and zinc ions. Zn^2^⁺ ions have intrinsic anti-inflammatory qualities in this system, whereas Cu^2^⁺ ions cause Fenton-like processes that prolong chemodynamic therapy.

Notably, the authors discovered an ideal copper to zinc ratio of 5:5. When exposed to external ultrasound stimuli and endogenous hydrogen peroxide, this particular compositional balance, along with carefully controlled atomic arrangements, strengthens piezoelectric polarization responses, enhances redox cycling, and synergistically increases ROS production. The system's ability to use both chemical and physical channels for therapeutic effect is demonstrated by these improvements, which are shown in **(Figure 9A)**.

Furthermore, the hydrogel alters immune responses by stimulating macrophage polarization, decreasing inflammation and encouraging regeneration. The hydrogel can adhere tightly to the skin and activate on demand, which can effectively kill germs and accelerate the skin healing in drug-resistant burn infections. This crystalline enhancement together with reduced lattice parameter indicates that balanced Cu-Zn inclusion is favorable for long range structural ordering. The first-principle calculations theoretically support the experimental observations shown in **(Figure 9B)**. The data suggest that the coherent lattice ordering prefers the stabilization of the Ti-Cu-Zn-Ti atomic configuration into Cu_5_Zn_5_@BTO and the minimized unit cell volume. This periodic arrangement of atoms leads to a symmetrical coordination environment around the titanium centers, thereby contributing to a more robust perovskite lattice framework and enhanced crystallinity. Conversely, any deviation from the ideal Cu/Zn ratio will result in a breakdown of this balance, which may cause lattice distortions and result in partial amorphization and/or loss of structural integrity. The incorporation of the elements Cu and Zn into the BaTiO_3_ lattice was further confirmed by elemental analysis as shown in **(Figure 9C)** which agreed with the XPS data. Using TEM, the authors were able to image these nanoparticles and see that they had a variety of shapes, including rectangular and spherical, and had a size range of approximately 5-7 nm, which is consistent with their nanoscale size. In order to evaluate the therapeutic potential of Cu_5_Zn_5_@BTO nanoparticles, the ROS generation was checked under both piezocatalytic and chemodynamic conditions. The ultrasonic stimulation creates a piezoelectric polarization effect in the nanoparticles that results in efficient charge carrier separation. The carriers approach the surface of the nanoparticle and interact with water molecules to create the formation of reactive hydroxyl (•OH) and superoxide (•O_2_^-^) radicals **(Figure 9D)**.

This mechanistic pathway was supported by electron paramagnetic resonance (EPR) spectroscopy in the presence of DMPO as a spin-trapping agent. In aqueous media, strong DMPO–•OH signals are observed **(Figure 9E)**, while in dimethyl sulfoxide (DMSO) the DMPO–•O_2_⁻ signals are predominant **(Figure 9F)**. In particular, the ultrasound treated samples show strong and characteristic EPR signatures of these radicals, while the control groups (no ultrasound exposure, no Cu/Zn doping) show low signals. This enhancement in generation of ROS is due to improved piezoelectric properties and beneficial band structure modifications induced by Cu/Zn co-doping.

In addition, the Cu_5_Zn_5_@BTO nanoparticles exhibited great antibiofilm activity, which is highly relevant for the treatment of chronic infections where biofilm formation prevents healing. The Cu_5_Zn_5_@BTO has shown better inhibition rate of methicillin-resistant Staphylococcus aureus (MRSA) biofilm development than Cu_2_Zn_7_@BTO and Cu_7_Zn_2_@BTO compositions upon exposure to ultrasound. Crystal violet staining further corroborates this inhibitory effect, demonstrating that the biofilm biomass of Cu_5_Zn_5_@BTO-treated samples is the lowest among the tested groups **(Figure 9G)**.

Immunofluorescence staining of wound tissues for arginase-1 (ARG1, the M2 phenotype marker) and inducible nitric oxide synthase (iNOS, the M1 phenotype marker) was further performed to clarify the immunological mechanism for the observed therapeutic efficacy. The analyses show a significant change to the anti-inflammatory M2 phenotype in tissues treated with Cu_5_Zn_5_@BTO under ultrasound stimulation **(Figure 9H)**. Specifically, this group shows a marked decrease in the number of iNOS-positive (M1) macrophages and an increase in ARG1-positive (M2) macrophages compared to the control samples. As a supplementary staining, CD86, another marker linked to the M1 phenotype, was immunohistochemically stained. In the Cu_5_Zn_5_@BTO + ultrasonic group, it demonstrated the comparable reduction in expression **(Figure 9I)**. These results collectively imply that Cu_5_Zn_5_@BTO causes macrophage polarisation to change from a pro-inflammatory to a pro-regenerative phenotype, which is essential for tissue repair and wound healing. While piezoelectric action therapy is a promising treatment for bacterial infections and inflammation, it is primarily theoretical for treating periodontitis and would require much more thorough testing and confirmation.

Periodontitis is not simply a localized bacterial infection but instead a chronic inflammatory condition which is caused by an abnormal host-microbiome relationship in a very diverse periodontal pocket environment. It may be too early to be hopeful that mechanically-generated ROS and electrical stimulation alone would be effective at disrupting mature oral biofilms and thereby alleviate underlying inflammation in the mouth, given that the outer matrix of these multispecies biofilms will make them difficult to disrupt. Another argument against piezoelectric activation is the unpredictable and sporadic nature of occlusal forces, as well as doubts on its ability to maintain activation *in vivo* for long enough to obtain a therapeutic effect. Let alone, little is known about their performance in an continuous challenge of saliva, enzymatic degradation and pH changes, which are all key factors in the development of periodontal disease. In addition, the long-term consequences of chronic low-level electrical stimulation on the periodontal tissue are not known, including what effects this may have on immune cells, fibroblasts and osteoblasts. If these physiological and mechanical issues are not resolved, the use of piezoelectric platforms in periodontitis may be overestimated. It is crucial to remember that rigorous antimicrobial testing, long-term safety data, and ongoing research in clinically relevant disease models will be necessary for the real clinical translation.

### 6.3 Piezoelectric regulation of ROS, immunity, and bone regeneration

Piezoelectric biomaterials are becoming multifunctional bioactive materials that can regulate redox chemistry, cell signaling, and tissue remodeling by directing the conversion of mechanical input to electrical output both spatially and temporally.

Recent studies have revealed that these materials have the ability to modulate intracellular ion transport, not only for ROS production but also for cell proliferation, differentiation and more broadly for regenerative programming 187. These systems can be intentionally engineered to include functionality of pro-regenerative signaling and control of oxidative stress by the inclusion of functional dopants, such as cerium for buffering ROS or magnesium- and calcium-based frameworks for osteoinductive support. In parallel, polarization by mechanical forces has been demonstrated to influence immune function, for example, to shift macrophages towards pro-healing phenotypes and to suppress chronic inflammatory pathways effectively, combining immune modulation and tissue repair. This is particularly useful for complex pathological situations where infection, inflammation and tissue degeneration are all present, such as piezoelectric platforms. Periodontitis is very similar: persistent dysbiosis, continuous inflammatory burden, and progressive alveolar bone loss, all of which coordinated piezoelectric mechanisms have the potential to impact simultaneously and at the same time 188,189.

In order to replicate natural signals, bioelectric modulation has been created as a unique way to control stem cell destiny, tissue regeneration, and immunological homeostasis. The loop of osteoinhibitory feedback from inflammation can be broken and the regeneration environment remodelled by electrically activating an external defect in the inflammatory one. Crucially, because bone is a piezoelectric material, its alveolar bone has a mechano-electrophysiological nature that makes it susceptible to electrical stimulation. BaTiO_3_, ZnO, and PVDF have recently been shown to be promising materials for the development of wireless, non-invasive piezoelectric materials with uses in inflammation management and bone regeneration. However, developing an effective piezoelectric platform for periodontal application requires simultaneously satisfying a number of difficult requirements, including high piezoelectric power, electrical signal transmission efficiency, true adaptability to the intricate and diverse periodontal microenvironment, and coordinated immunomodulatory and osteogenic effects.

There is no existing material that fulfills all these requirements singly 190. For instance, PVDF has good mechanical flexibility but is not bioactive and osteoconductive, which is required for adequate bone integration. However, BaTiO3 has high piezoelectricity but the inherent stiffness is a problem for soft periodontal tissue integration. The improvement of a single performance factor is not likely to translate into clinical benefit, and an integrated approach to material design is needed for clinical use 191.

Among these potential materials, ZnO is highlighted for its excellent biocompatibility and fabrication process that is well-controlled. However, conventional ZnO nanoparticles have been found to be less piezoelectric due to free charge carriers in the bulk that move in the internal electric field, and thus screen the output signal, thus reducing the output signal. A solution is provided by heterojunction engineering which creates internal electric field at the heterointerfaces to increase the charge separation and carrier directional transport and to reduce the electron-hole recombination. Moreover, the selective trapping of free carriers via electron-trapping sites at the interface, for example, by introducing such defects, enables further control, namely interfacial defect engineering. Nonetheless, using conventional fabrication techniques, lattice mismatches and interfacial resistance are introduced, and the overall piezoelectric performance is reduced by these advantages 192-194.

In recent years, the efforts have been directed towards the synthesis of ZnO/ZnS heterojunction in-situ to enhance lattice compatibility and interface quality. This technique reduces charge screening effects and facilitates effective electron conduction across surfaces. In order to increase the mobility of the carriers by creating electron traps at their interfaces, a manufacturing process utilising sulphur substitution and annealing has been devised. Defect engineering and heterojunction-induced electric fields work together to provide a two-stage enhancement process that greatly improves the composite system's piezoelectric sensitivity 195. The electrical transmission effectiveness of the electrical stimulation in the anatomically complicated, spatially limited periodontal lesion is a difficulty in addition to material design.

Such a requirement requires a matrix that allows for adequate electrical conductivity and can be compatible with the natural biological surroundings. The investigations of the use of hydrogels, made from polyvinyl alcohol (PVA) have been done, because they are biocompatible, biodegradable, having three-dimensional network structure suitable for targeted drug delivery. Traditional PVA hydrogels, however, suffer from intrinsic low ionic conductivity, low injectability and limited tunability for non-circular defects that impede efficient electrical signal propagation across the defect site. Previously, it was demonstrated that the Hofmeister series of ions can affect the hydrogen bonding of PVA networks in ways that enhance their mechanical strength and ionic conductivity 196-198.

According to this, Zhou *et al*. 199 suggested using magnesium chloride (MgCl_2_) and sodium citrate (Na_3_Ct) as ion sources to establish a dual-salt system in a PVA hydrogel matrix. In addition to improving multivalent coordination and ionic interactions, this dual-salt method strengthens the polymer network and raises ionic conductivity. As a result, we are better able to inject and adjust to the periodontal flaws' uneven geometry. In addition to its immunomodulatory and bone-regenerating effects, magnesium ions (Mg^2^⁺) also have other bioactive characteristics, such as influencing macrophage polarization state and triggering important osteogenic signaling pathways.

This composite material simultaneously enhances the piezoelectric property and exhibits excellent mechanical integrity, electrical conductivity and injectability. In this way, it can make it possible for the mechanical, electrical, and immunological dynamics of the periodontitis microenvironment to be synchronized. Therefore, this integrated strategy further advances the potential for successful periodontal regeneration treatments by providing a designed platform that can be bioadaptive and may directly promote osteogenesis, with a simultaneous immunological remodeling at the lesion site.

The piezoelectric property of ZnO was improved by the researchers with a dual-strategy modification approach **(Figure 11A)**. In the pristine ZnO, the internal piezoelectric field induces the migration of excited charge carriers which accumulate at one end to form a screening layer that reduces the total electrical output. On the other hand, the ZnO/ZnS heterojunction has a type-II band alignment at the interface due to the energy level difference between the two materials. This alignment drives electrons from ZnO to ZnS, reducing charge accumulation and suppressing the screening effect. Furthermore, the annealing process and partial substitution of oxygen with sulphur create interfacial oxygen vacancies. These vacancies act as electron traps which allow for more efficient charge transport. The lattice fringes obtained in high-resolution transmission electron microscopy (HRTEM) images of both ZnO and ZnS are distinctly observed and confirm the formation of a well-defined heterojunction interface **(Figure 11B)**. EPR analysis indicates that the oxygen vacancy signal is the strongest in the ZnO/ZnS-0.04 sample, indicating that the vacancy is formed by a combination of annealing and *in situ* sulfidation, while the substitution of sulphur further facilitates the formation of defects **(Figure 11C)**. Piezoresponse force microscopy (PFM) measurements show that ZnO/ZnS-0.04 has significantly higher piezoelectric response than pure ZnO or ZnS **(Figure 11D)**. The results show that the *in situ* synthesised ZnO/ZnS-0.04 heterojunctions enhance the piezoelectric properties due to the synergistic effect of interfacial coupling and defect engineering and have potential for bioelectrical stimulation in periodontal applications.

The composite piezoelectric hydrogel was applied to a rat model of chronic periodontitis to assess therapeutic efficacy. They injected different hydrogel formulations into periodontal defect sites and evaluated healing after four weeks **(Figure 11E)**. The periodontal probing values demonstrate the presence of deep periodontal pockets greater than 1 mm at mesial and distal sites in the control group, showing severe tissue damage **(Figure 11F)**. Tight gingival attachment and a significantly shallower probing depth, with effective tissue repair was noted in the POG-HC treated group, however, this was not seen in the other group. In addition, the results of the micro-CT analysis revealed a better alveolar bone regeneration for the treated groups. Histological analysis was performed using H&E staining to evaluate tissue architecture, Masson's trichrome staining to assess collagen organisation, and TRAP staining to assess osteoclast activity **(Figure 11G)**; both osteogenesis and immune regulation were important to consider when evaluating periodontal healing. It is an integrated system that consists of multifunctional hydrogel platform and a dual-modification strategy to address the fundamental challenge of the conventional piezoelectric materials. The Mg^2+^/citrate dual-salt network provides a better ionic conductivity and mechanical stability at the same time and is more osteogenic. The multinetwork hydrogel architecture allows it to be injected and is stable in difficult periodontal conditions. Meanwhile, the ZnO/ZnS heterojunction, enhanced by interfacial and defect engineering, significantly promotes charge separation and piezoelectric output. These properties make them suitable for efficient electrical stimulation, immune modulation and bone regeneration in periodontal therapy.

Although mechanical devices story is still oversimplified and inadequately supported, the heterojunction technique is theoretically intriguing. Although oxygen vacancy engineering and type-II band alignment are used to reduce charge screening and increase piezoelectric output, there is insufficient evidence to support the direct conversion of these interfacial electronic effects into significant bioelectrical stimulation under physiological conditions. The idea that improved nanoscale charge separation may persist and function well in the highly dynamic, ion-rich periodontal environment is particularly questionable. Moreover, oxygen vacancies are solely seen as beneficial electron traps; their potential instability, susceptibility to recombination, and long-term impacts on material deterioration and biocompatibility are not covered. Additionally, the biology data is terribly insufficient for credible regeneration claims. Only a few aspects of the gradual pathological process of chronic periodontitis, which is marked by ongoing tissue degradation, complex biofilm composition, and inflammation, are replicated in short-term rat models. There is insufficient quantitative data to support the conclusions regarding probing depth and histological appearance, including accurate bone morphometry, inflammatory cytokine profile, and a reliable treatment standard.

Additionally, the system includes several functional components, some of which are the hydrogel matrix, the ionic conductive network, and heterojunction, which cannot be separated to evaluate the individual contribution of each component, thus preventing the verification or improvement of the claimed synergistic advantages.

The relevance of this work is also exaggerated for translation. Substantially different *in vivo* performance in real periodontal tissue can occur due to saliva exposure, enzymatic activity, microbial attack, and continuous mechanical stresses. All of this makes it difficult to see how the increased piezoelectric response seen under controlled conditions would persist over time, or if it would be able to generate the signals of bioelectricity required to stimulate tissue regeneration over time. In the absence of biofilm resistance, long-term stability, and functional durability, the device is an interesting materials-design exercise more than a convincing alternative device for the treatment of periodontitis.

### 6.4 Piezo-sonodynamic therapy and immunotherapy

The major problem in the treatment of periodontitis is the elimination of deep tissue bacterial infection. Standard methods such as topical antibiotics and mechanical cleaning can provide some improvement in the case of infection, but bacteria in the deep periodontal pockets may be inaccessible 200,201. This allows bacteria to grow in these concealed areas and cause repeated inflammation and make treatment difficult. Stronger and more specifically targeted antimicrobial strategies are therefore required. Anaerobic bacteria make around 70% to 90% of the microbial flora on the surface of teeth in periodontitis, and their numbers are significantly higher in subgingival plaque 202. A crucial element in breaking the vicious cycle that might worsen periodontitis is the idea of anti-inflammatory tactics. The host's inflammatory response to bacterial infection leads to the production of mediators including TNF-α and IL-1β, which cause alveolar bone resorption and connective tissue degradation.

Active gas molecules like nitric oxide (NO) are good at penetrating tissue, making gas therapy especially potent for treating infections. One major benefit of this treatment is that it can be used to target hard-to-reach areas of subgingival biofilm, increasing the effectiveness of the treatment. Other gases also have anti-inflammatory properties by repressing the production of inflammatory factors or by directly affecting immune cell functions. NO in particular has broad spectrum bactericidal properties that disrupt the structure of bacterial biofilm, with the capacity to interfere with microbial energy metabolism, therefore it has unique properties on the treatment of periodontitis. Research has also shown NO plays a role in controlling inflammation by blocking NF-κB signaling. Therefore, an effective bactericidal strategy based on externally triggered, localized, explosive release of NO was investigated in this study, which also utilized the favorable NO pharmacokinetics, such as its longer half-life (on the order of minutes) and greater diffusion radius than ROS.

One potential approach is to use the sustained diffusion characteristics and the prolonged effect of NO, with US turned off, under low concentration conditions to have a long-lasting anti-inflammatory effect. This approach develops a treatment paradigm that is "dynamic and precise regulatory. L-arginine (l-arg) is a good source of NO, which is generated by oxidative deamination. A number of variables, like as pH, oxidative stress, and mechanical stimulation, affect its release. Therefore, creating a carrier that responds to environmental cues to transport NO to the infection site in a spatiotemporal manner should improve the synergy and maximise the antibacterial and anti-inflammatory effects 205.

Due to its deep tissue penetration and noninvasive nature, SDT is considered the best choice for local periodontal treatments. The large specific surface areas and porous architectures of titanium-based organic frameworks (Ti-MOFs) set them apart as effective sonosensitizers. By arranging metal nodes with organic ligands in a fashion that resembles the lattice structure of semiconductor materials, an ordered channel for electron movement is created, significantly increasing the efficiency of electron transport 206. However, the intrinsic flaws of the metal nodes, such as unsaturated coordination sites and a high electron-hole recombination rate, limit the efficiency of ROS formation. Fortunately, the polarity of the material is significantly increased by using a Mn-doping technique to produce a mixed Mn^2+/^Mn^3+^ valence state. The Mn doping-induced piezoelectric effect spontaneously polarizes under mechanical stress to produce an intrinsic electric field, which further promotes the generation of ROS. This bimetallic dual advantage significantly increases SDT's therapeutic effectiveness while also making up for the structural shortcomings of Ti-MOFs 207-209.

Future studies should evaluate ultrasound-responsive and piezoelectric antimicrobial therapies against physiologically relevant multispecies anaerobic biofilms rather than relying primarily on single-pathogen or planktonic models. Particular attention should be given to EPS-mediated protection, saliva-derived biofilm architecture, bacterial recolonization, and the preservation of oral microbiome balance. More representative multispecies, ex vivo, and longitudinal *in vivo* models will therefore be essential for determining whether these strategies can achieve sustained dysbiosis control without causing nonspecific disruption of the oral microbial community.

In order to alleviate bacterial dysbiosis, Li *et al*. 210 proposed a bifunctional nanoplatform. The boric acid esterification technique was used to accomplish targeted enrichment and selective eradication of pathogens. Concurrently, l-arg is added as a NO donor to release NO when ROS produced by SDT are stimulated, preventing excessive inflammatory reactions. This nanoplatform combines the deep tissue penetration capacity of SDT, the anti-inflammatory properties of NO gas, and the precision targeted bactericidal function into a single framework **(Figure 12A)**. (1) Biofilm production is significantly reduced when periodontal infections such Porphyromonas gingivalis are precisely eliminated. (2) NO reduces soft tissue inflammation and alveolar bone loss by regulating the production of inflammatory proteins, preventing the formation of the NLRP3 inflammasome, and suppressing the activation of the NF-κB pathway. The efficiency of SDT was increased by the piezoelectric enhancing effect of Ti/Mn-MOFs.

Ti/Mn-MOFs were successfully produced using a one-step hydrothermal method, with an average particle size of around 190 nm. High-angle annular dark-field scanning transmission electron microscopy (HAADF-STEM) was used to further examine the structural properties of Ti/Mn-MOFs. The homogenous distribution of O, N, C, Ti, and Mn was shown by elemental mapping analysis, confirming the successful synthesis of the Ti/Mn-MOFs **(Figure 12B)**. Piezoresponse force microscopy (PFM) was used to characterize the piezoelectric characteristics of the Ti/Mn-MOFs. Atomic force microscopy (AFM) was used to further investigate the morphology of the Ti/Mn-MOFs. It was found that the thickness was around 25 nm. Ti/Mn-MOFs were photographed using out-of-plane PFM phase and amplitude **(Figure 12C)** Ti/Mn-MOFs showed both a phase-voltage profile that matched the voltage-electromagnetic hysteresis loop and a distinctive butterfly loop amplitude–voltage curve. Up to 156.21 pm V^-1^ is the computed piezoelectric coefficient d33. This might be because the Ti/Mn-MOFs include Mn-Ti-oxo cyclic octamers as SBUs due to the incorporation of manganese into titanium-based secondary building units (SBUs). An established method for examining deep periodontal pocket-associated pathogenic processes, such as dental plaque, inflammation, and bone resorption, as well as assessing systemic/noninvasive treatment strategies, is the Sprague-Dawley (SD) rat periodontitis model. In order to assess the *in vivo* therapeutic effectiveness of Ti/Mn-MOFs-P-L against periodontitis, a rat periodontitis model was created via silk ligation **(Figure 12D)**. In particular, a bacterial solution was administered next to the ligature site to cause inflammation after a 5-0 silk suture was placed around the upper palate's second tooth. For 14 days, rats with experimental periodontitis received twice-daily intrasulcular injections (200 μL/site) at ligature sites. Both two-dimensional imaging data on the cross-sectional root furcation region of the maxillary second molar and three-dimensional reconstruction pictures of the rat maxilla were effectively obtained **(Figure 12E)**. A new sonosensitizer Ti/Mn-MOFs core integrated with PBA targeting ligands and l-arg NO donors to create a multifunctional therapeutic agent is a promising technique. Through covalent interaction with bacterial cell walls, the PBA allows for specific bacterial targeting. ROS is produced to oxidize l-arg during US stimulation, resulting in regulated NO release. More significantly, NO has anti-inflammatory and cooperative antibacterial properties. Its prolonged half-life makes it easier to concentrate for bacterial elimination during US activation and inhibits the formation of NLRP3 inflammasomes to produce anti-inflammatory effects following US cessation. Furthermore, Mn doping optimizes the SDT efficiency by introducing defect energy levels that lower the energy needed for electronic transitions. This will effectively reduce the band gap of the Ti-MOFs and induce piezoelectric polarization when subjected to mechanical strain.

The triple application of SOT, nitric oxide delivery and piezoelectric enhancement in a single nanoplatform to overcome the multifactorial nature of periodontal disease is a forward-looking approach, but still a number of important issues must be addressed before its full potential can be realized. Future study should be directed towards the precise spatiotemporal control of the generation of ROS and NO, to allow their antibacterial activity without damage to the surrounding periodontal tissue. To validate the effectiveness of targeted strategies such as boric acid functionalization, it is important to use more clinically representative multispecies biofilm models instead of simplified test systems. It should be noted that in addition to the proposed mechanisms of increasing sonodynamic efficacy, namely Mn doping and piezoelectric effects, their specific role still requires quantitative confirmation under physiologically realistic ultrasound conditions, especially regarding the effect of ion leaching, long-term stability of the material and its ability to maintain function in the oral environment.

From a translational perspective, it will be crucial to go beyond short-term animal models in order to evaluate the long-term sustainability of treatment results, such as the avoidance of biofilm recurrence and the preservation of alveolar bone integrity. The necessity for more useful and patient-friendly delivery methods, including adhesive or implantable platforms with extended retention and on-demand activation, is further highlighted by the existing reliance on recurrent local injections. Furthermore, in order to uncover distinct molecular pathways and optimize treatment design, future research should focus on deciphering the individual and combined effects of targeted ligands, NO release, and piezo-enhanced sonodynamic activity. These limitations need to be solved in order for such multifunctional nanoplatforms to progress from intriguing experimental constructs to clinically viable periodontitis therapy alternatives.

### 6.5 Ultrasound imaging in periodontitis

The 2017 World Workshop on the Classification of Periodontal and Peri-Implant Diseases and Conditions defined "periodontal phenotype" as an umbrella term covering gingival phenotype, bone morphotype, and tooth dimensions within the framework of mucogingival deformities 211. Gingival phenotype is characterized by two components: soft tissue thickness (STT) and gingival width. Bone morphotype, by contrast, refers specifically to crestal bone thickness (CBT). A further clinically relevant measure is soft tissue height (STH), representing the apico-coronal span of the dentogingival complex that is, the supracrestal tissue attachment (STA) combined with sulcus/probing depth. In clinical practice, the measurement of STH is made from the free gingival margin to the crestal bone 212.

In clinical practice, the identification of gingival phenotype usually is subjective 213. A popular method is to place a periodontal probe in the sulcus and determine if it can be seen through the overlying tissue (thin) or not (thick). 214 This binary method does not however, consider intermediate thickness values. Direct STT measurement can be performed with alternative instruments, such as calipers, endodontic files, and needles, but these methods are invasive and can cause complications, including misangulation of the instruments, rubber-stop slippage, and need for local anesthesia 215. The assessment of STH is even more challenging, as there is currently no non-invasive method available: both methods that are accepted (bone sounding and direct measurement after flaps are reflected) require penetration of the tissue 216. Standard 2D radiographs are not sufficient to assess CBT because they are unable to provide a true assessment of the thickness of crestal bone and facial bone. Cone-beam computed tomography (CBCT) is considered to be an acceptable alternative in clinical practice, but may be limited by the risk of radiation exposure and costs especially for patients undergoing repeated imaging over time 217.

Increasingly new imaging techniques are being investigated to fill these diagnostic gaps, including ultrasonography 218. Ultrasound is an intriguing way to see the periodontal structures since it employs sound wave reflection and is non-invasive and non-ionizing 219.

Several cadaver studies, both human and porcine, have shown that ultrasound has the potential of providing very accurate measurements of periodontal dimensions, and recent investigations have reported acceptable inter-reader agreement for interpretation of images from such measurements 220. All of these pieces of evidence suggest that ultrasound is a relatively reliable and repeatable means to measure periodontal tissues. The *in vivo* ultrasound performance, however, remains to be validated with respect to histology as the standard *in vivo* technique for these measurements, which is still considered the gold standard 221.

Kripfgans *et al*. 222 investigated the accuracy of ultrasound (US) in measuring the dimensions of inflamed periodontal tissue for seven parameters using three assessment methods, ultrasound, CBCT and histology. They found excellent-to-strong agreement compared to histology for several measures bone level, bone thickness, soft tissue thickness at 1 mm, soft tissue height, and gingival recession. In particular, ultrasound was better than CBCT in cases with thin crestal bone. The physics behind ultrasound imaging is founded on the interaction of the ultrasound waves with tissue; waves scatter or reflect depending on the acoustic impedance and physical dimensions of the tissue that they come in contact with. The reflected sounds are picked up by a transducer and are turned into an image. Structures smaller than the acoustic pulse size diffract the waves in many directions, larger ones reflect the waves according to Snell's law 223. To illustrate, a 24 MHz transducer is able to resolve objects as small as 61.7 μm in water, but in the oral tissues, small-scale features are often clinically significant yet not resolved. Resolution, then, is one of the most important limitations in the performance of transducers, and is dependent in large part on beam geometry and center frequency. The axial resolution is actually specifically the half of the spatial pulse length, which is the product of the number of cycles per pulse and the wavelength 224. The higher the center frequency, the shorter the pulses and the better the axial resolution.

Jokerst *et al*. 225 constructed a 128 element, 40 MHz transducer with a small footprint, which allows for full arch imaging with third molars. They measured the spatial resolution at different depths with a line spread function obtained from a Nichrome wire phantom and explored the effect of transducer tilt (roll, pitch and yaw) on the image quality of posterior teeth. To validate its capacity to measure soft tissue measurements (gingival thickness and gingival height), they took 14 excised swine teeth with attached gingiva and compared the images with transgingival probing directly on the resected tissue **(Figure 13A-B)**.

The best resolution performance was obtained for 7 mm focal depth where the lateral resolution was optimum. From this depth, targets in the form of circles started to show elongation, and the greater the distance the more pronounced **(Figure 13C)**. Circularity values were recorded at seven depths, 2.2, 4.4, 6.6, 8.8, 11, 13.2, and 15.4 mm yielding results of 0.456, 0.676, 0.912, 0.648, 0.532, 0.431, and 0.345, respectively. The highest circularity was measured at 6.6 to 7 mm suggesting little shape distortion, while the lowest value of (0.345 at 15.4 mm) suggested significant elliptical distortion. This trend is consistent with the fact that the lateral resolution gets worse as we go deeper. Similarly, error margins, obtained from repeated measurements over a range of focal depths from 3-16 mm, confirmed this pattern, with errors of only ±30.72 μm at the near ideal depth of 6.6 mm and ±81.59 μm at the far end of the depth at 15.4 mm, illustrating the critical importance of imaging at the near ideal depth of 7 mm for measurement accuracy.

Two blinded image interpreters independently identified the anatomical landmarks to measure the ultrasound-based gingival height (iGH) and thickness (iGT). The measurements they took were very close together around that line of equality (y = x). There was excellent inter-examiner agreement for both iGH (r = 0.9692; p < 0.0001) and iGT (r = 0.6695; p = 0.0003). This notwithstanding, the inter-examiner standard deviations were small (0.03 mm for iGH and 0.08 mm for iGT) compared to the inter-examiner standard deviations previously reported in clinical use.

In the present part of the study, the mandible of a pig was used which had three molars and three premolars, photographed from the buccal and lingual sides, respectively **(Figure 13D)**. Results showed significant thickening of the gingiva around the molars than the premolars, most significantly at the lingual aspect. The mean gingival thickness was found to be 0.78 ± 0.14 mm at premolar sites and 1.14 ± 0.35 mm at molar sites. Imaging both buccally and lingually with the high resolution PL57x_1 128 transducer allowed clear differentiation and quantification of soft tissue characteristics throughout the jaw.

To date, there is still a heavy reliance on mechanical disruption of biofilm through scaling and root planing (SRP), which continues to be the accepted clinical gold standard for treatment of periodontitis. However, complete eradication of pathogenic bacteria is challenging in difficult to access pockets, furcation involvement, and intrabony defects where there is persistent infection and recurrence. Therefore, materials that respond to ultrasound have been developed as a complementary approach, in addition to conventional therapy. These materials can be activated by external ultrasound exposure to produce reactive oxygen species (ROS) which can enter tissue defects and release therapeutic payloads or can be activated individually to promote tissue repair and collectively enhance antimicrobial activity. SRP, refractory periodontitis, deep intrabony defects, and peri-implantitis are some of the conditions that are often not treated effectively in the long term and in this regard the kind of multifunctionality that this treatment brings can be particularly useful. However, there is still hope for meaningful translation, as there are limited issues that remain to be addressed: retention of materials in periodontal pockets, lack of standardization of ultrasound protocols, long-term safety issues and regulatory challenges of combined materials and devices products. Even so, ultrasound-responsive therapy represents an appealing precision-medicine strategy offering localized activation, minimal systemic exposure, and the capacity to simultaneously tackle microbial imbalance, inflammation, and tissue regeneration.

## 7. Clinical workflow

The variability of the mechanical environment of the oral cavity in terms of the intensity, frequency, length and number of masticatory forces experienced at various locations and from person to person is significant and must be taken into consideration in the design of future chewing- and occlusion-based oral therapeutic devices. As such, the therapeutic output of self-powered piezoelectric devices needs to be quantitatively linked to physiologically realistic mechanical loading in order to build reproducible dose-response relationships. Extended *in vivo* studies are also required to evaluate device fatigue, structural durability, the consistency of device piezoelectric output, and the duration of function when exposed to sustained mechanical forces and continuous oral biofilms, pH changes, and temperature changes. In addition, in cases of implantable or transient devices, it will be crucial to define the fate of the material following treatment (whether by controlled degradation, bioabsorption or safe removal), and to characterize the biological effects of any degradation products that are present. These factors will be essential to make reliable, durable proof-of-concept self-powered oral devices a reality. There is some preclinical data that supports the use of ultrasound responsive materials as a promising approach to combating bacteria, reducing inflammation and promoting tissue regeneration 226. Despite this, their usefulness will depend on their ability to fit in with current periodontal treatment. Periodontal therapy is more difficult than those used for cancer therapy or for the therapy of cutaneous wounds, because it has to deal with a constantly changing environment, in which there are multispecies biofilms, a continuous flow of saliva and gingival crevicular fluid, mechanical loading from mastication, and pockets that are inherently difficult to access. In addition to improved materials and devices, integration of these systems into existing periodontal care pathways will be a key factor needed to move toward clinical applications 227,228.

There is currently evidence-based guidance towards a sequential approach that includes biofilm control, risk factor management, and maintenance therapy. SRP should be the foundation of any other nonsurgical treatment and is the basis of any other nonsurgical treatment. The European Federation of Periodontology's (EFP) S3-level clinical guidelines recommend treatment goals of "elimination or reduction of periodontal pockets, control of inflammation, and prevention of further attachment loss by effective disruption of subgingival biofilms. For those sites that do not respond to first line therapy, further action is usually necessary, either in the form of additional treatment or regeneration procedures 229,230.

In this context, the use of ultrasound-responsive materials cannot be considered as substitutes for conventional therapy, but rather as complementing ones that can complement the limitations of conventional therapy. A realistic clinical pathway may start with a detailed periodontal assessment, clinical probing, radiographs, Bleeding on probing (BOP), Clinical attachment level (CAL) determination, and possibly microbiome profiling. These materials could be locally applied after SRP as grafts and injected as gels, particles, bioactive membranes, coatings or scaffolds 231,232. Targeted production of ROS, piezoelectric stimulation, controlled drug release, immune modulation or regeneration would then follow by ultrasound activation. By ultrasound activation, targeted production of ROS, piezoelectric stimulation, controlled drug release, immune modulation or regeneration would follow 38,39,233,234.

### 7.1 Residual biofilm control

One of the more short-term uses is handling biofim that remains following SRP. Full mechanical removal is not possible in all areas where there are deep pockets, root concavities, furcation areas and other anatomical areas where resistant biofilms occur, even after thorough mechanical debridement. EFP guidelines acknowledge that although pockets are often left by routine therapy, they may require additional treatment. The use of ultrasound-induced antimicrobial systems may be used as an adjunctive device targeting survivors of mechanical instrumentation 235,236.

Future studies should clearly distinguish between outcomes which have been experimentally verified and those which are merely potential outcomes when characterizing biomaterials that are responsive to ultrasound. These systems may in principle have antibacterial, immunomodulatory and osteogenic properties, but these properties have to be demonstrated in a single physiologically relevant system and not simply inferred from the results from separate, simplified experimental systems. Future studies should therefore focus on combined studies that investigate infection control and immune response, along with functional tissue regeneration, in clinically relevant multispecies and *in vivo* models in order to prevent exaggerated conclusions and the understanding of the true therapeutic potential of these materials.

### 7.2 Refractory periodontitis

Another potential application is the problem of refractory periodontitis, in which there is a persistent inflammatory reaction and reinfection with bacteria even after successful conventional treatment. This is because antibiotic courses are prescribed on a repeated basis generally not recommended due to the risk of developing resistance and the disruption of the microbiome. There are, however, materials available that can be activated by ultrasound to generate ROS at a specific site, thereby reducing the systemic exposure to the drug. The method could be especially beneficial for biofilm infections which are nonresponsive to conventional antimicrobial therapy 237, 238.

### 7.3 Regenerative periodontal therapy

These systems may also play a useful role in tissue regeneration, in addition to infection management. Alveolar bone loss is one of the major consequences of periodontitis which has great implications on tooth prognosis. Whereas some more advanced morphologies are clinically proven to be treatable with current regeneration techniques, such as guided tissue regeneration (GTR), enamel matrix derivatives, and bone grafting, there is still no uniformity and predictability of outcomes with these more advanced morphologies 239. The use of materials which generate localized electric signals when stimulated by mechanical energy offers a novel approach to inducing bone formation, angiogenesis and periodontal ligament repair, and controlling infection. In profound intrabony defects, where tissue regeneration and microbial control are critical for healing, this dual role will be especially important 240,241.

### 7.4 Setting meaningful therapeutic endpoints

To facilitate clinical translation, objectives must be well-defined and relevant. A mere antibacterial activity was not adequate to demonstrate; nonetheless, future investigation should examine more relevant clinical effects. Current periodontal guidelines identify success of periodontal therapy as pocket closure, a decrease in probing depths, no periodontal bleeding on probing, no worsening attachment loss, and a halt of disease deterioration. Specifically, good results usually equate to a decrease in periodontal pocket depth (PPD), an increase in clinical attachment level (CAL), decrease in BOP (blood on probing), decrease in inflammatory markers, and radiographically evident alveolar bone regeneration. Shallow pockets and few indices of periodontal disease activity (PDA) suggest stable periodontal health 242. Equally important is a clear definition of treatment failure. If the pockets remain deep, the bleeding has not subsided after probing, the inflammation continues, the bone has not regenerated, bacteria have re-invaded the area, and there has been persistent bone loss, it indicates that the therapy is not working. Failure of microbial control or inadequate modulation of host response also may be indicated by late relapse after initial improvement. Future studies should therefore include longer follow-up periods to determine the durability of effect, beyond the short-term antimicrobial effect.

### 7.5 Remaining challenges

Even with this much progress, however, there are a number of challenges that need to be overcome for the technologies to be adopted in a clinical setting. One of the main difficulties is keeping the material in the periodontal pocket for a sufficient time to have a therapeutic effect. The oral cavity is constantly subject to the ebb and flow of saliva, mechanical strain on the teeth from chewing and normal oral hygiene routines that can all cause therapeutic materials to be dislodged too early. In the future, designs of materials should therefore focus on adhesion, degradation and retention. One of the other challenges is the standardization of the parameters of ultrasound therapy. Therapeutic outcomes may be greatly influenced by various factors such as frequency, duty cycle, exposure time, cavitation characteristics, and energy distribution. Low amounts of ROS might result in insufficient antimicrobial action, whereas high ROS levels can damage healthy tissue. Therefore, before such therapies are employed in the clinic, standard treatment procedures and safety limitations will be crucial, particularly if they are expected to be repeated throughout long-term maintenance therapy 36,37,243.

Scalability, sterility, regulatory approval processes, and batch-to-batch consistency are other factors to take into account. The current systems for ultrasound-based control are more challenging to scale up and have a very complicated makeup. Furthermore, compared to conventional periodontal therapy, combination products which combine biomaterials with ultrasonic equipment may come under closer examination.

### 7.6 Looking ahead

The integration of cutting-edge biomaterial engineering, microbiome analysis, and precise ultrasound delivery has the potential to completely transform periodontal therapy and usher in a new age of individualized care. By altering the material composition, therapeutic payload, and ultrasonic settings, future techniques might be developed to address the disease's severity, microbial makeup, inflammatory condition, and regeneration needs. This development has the potential to significantly improve treatment predictability and is in line with the rest of precision periodontology.

In summary, ultrasound-responsive materials show promise as a platform for delivering antimicrobial treatment and tissue regeneration in periodontal tissues at the same time. However, thorough testing in a suitable animal model, standardized ultrasonography procedures, a thorough safety assessment, and carefully planned randomized controlled trials would be necessary for the clinical realization of this promise. These technologies are restricted to promising concepts in the lab and never reach the chairside for application in the treatment of periodontitis and other oral disorders in the absence of these sorts of comprehensive validations.

## 8. Conclusion

Ultrasounds are a possible therapeutic option for periodontitis, a complicated gum disease that is known to be linked to a number of events, including oxidative stress, bacterial community dysbiosis, immunological dysfunction, and bone destruction. Because ultrasound can penetrate tissues and non-invasively activate functional materials that can convert mechanical or sound energy into chemical or electrical output, this technique is highly fascinating. Additionally, the sonodynamic and piezoelectric materials may work in concert to break the biofilm by controlling the production of reactive oxygen species (ROS) and piezoelectric polarization, which is involved in immunological regulation, redox, and signaling pathways related to bone regeneration.

Crucially, delivery systems (such hydrogels, membranes, and scaffolds) lack intrinsic ultrasonic responsiveness and are passive matrices that may be employed to localize and stabilize active components. In difficult oral settings, they aid in better retention, targeting, and treatment. Moving progress in this field should entail transforming earlier proof-of-concept concepts into mechanistically sound and therapeutically feasible systems. This involves adjusting material responsiveness and ultrasonic settings for therapeutic application that is both safe and effective. There are some issues that should be addressed in the areas of increased research demands for oral biofilm recurrence, long-lasting stability in the mouth, and integration with therapeutic systems.

The gap between mechanistically sound and clinically useful periodontal therapeutics using ultrasound must be bridged for the implementation of the ultrasound responsive techniques into clinical practice.

## Figures and Tables

**Scheme 1:**
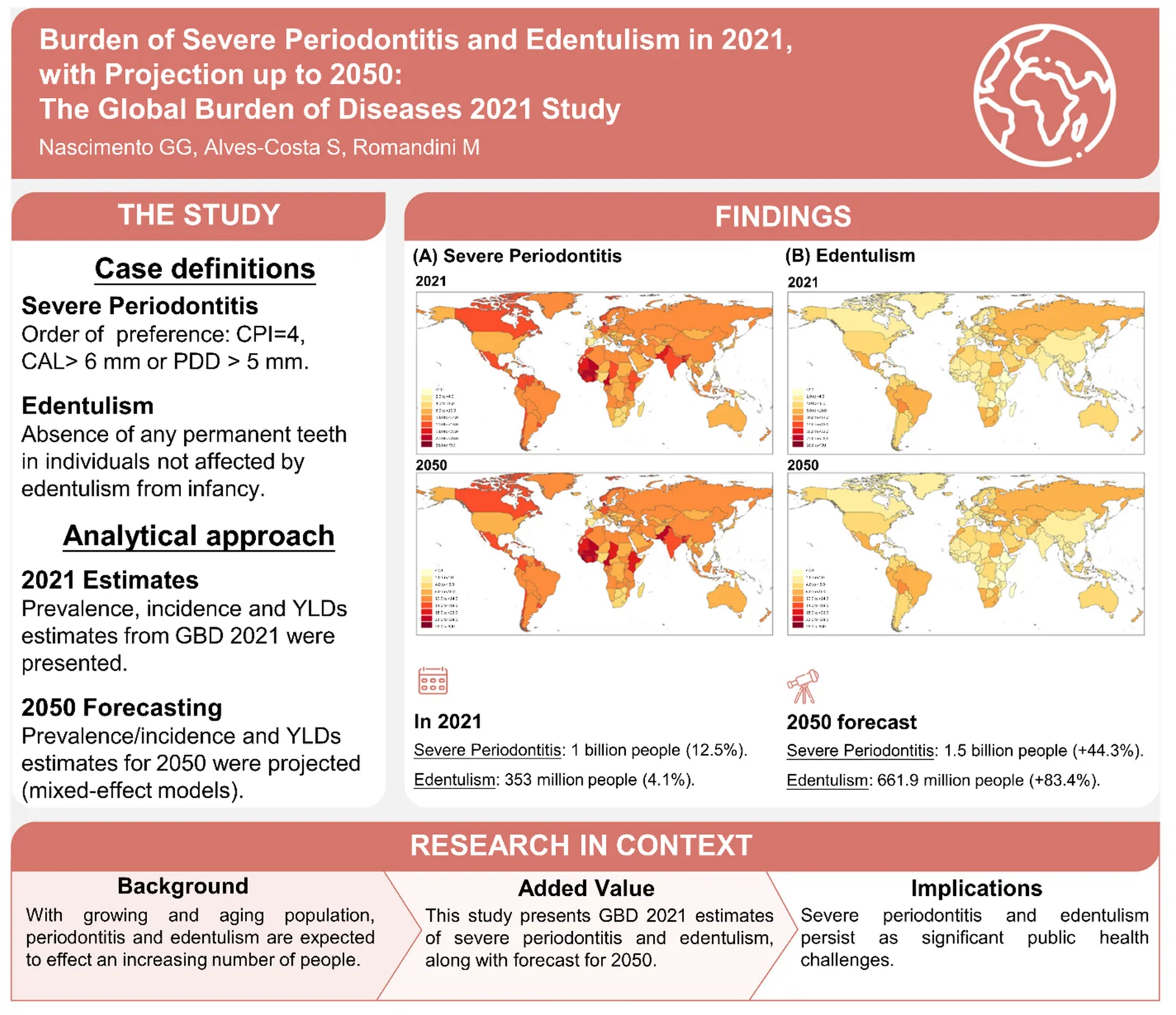
Forecasted prevalence of severe periodontitis and edentulism worldwide by 2050. Adapted with permission from ref. 3, Copyrights 2024 WILEY.

**Scheme 2:**
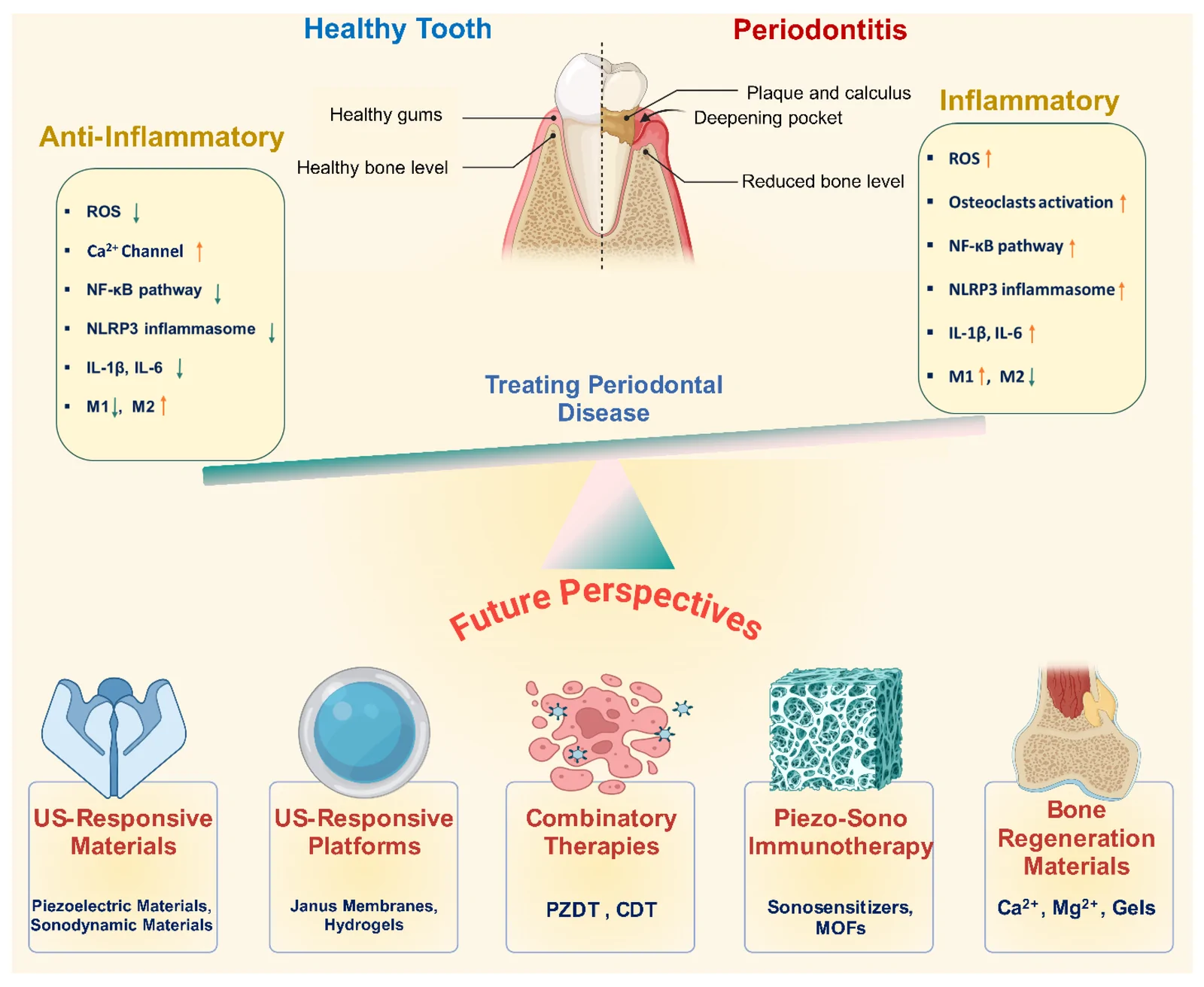
An overview of the mechanisms of periodontitis and potential treatments in the future. The figure was designed using BioRender.

**Scheme 3:**
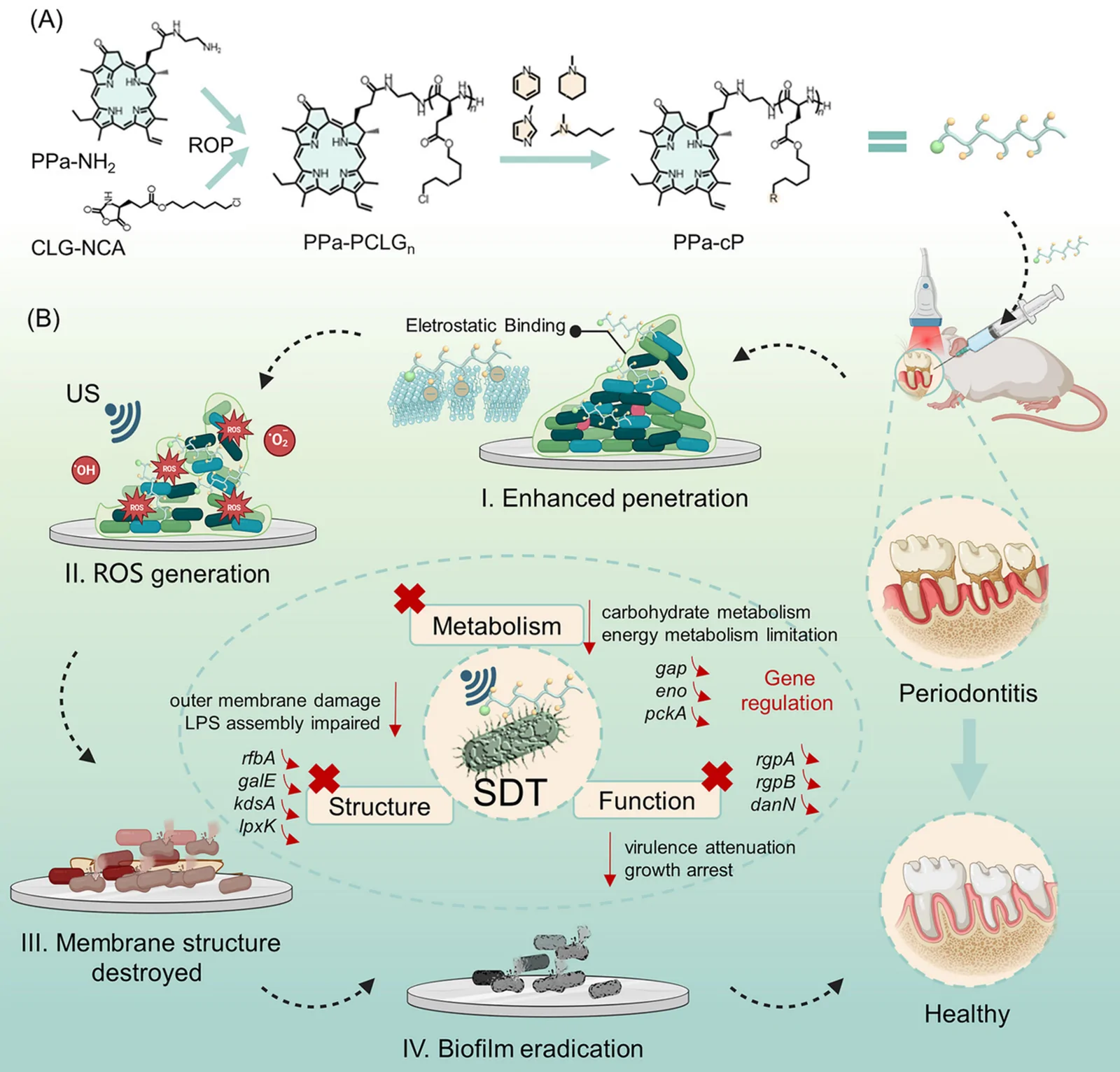
Schematic Illustration of the Antibiofilm Mechanisms and Therapeutic Processes Underlying Periodontitis Treatment. Adapted with permission from ref. 96, Copyrights 2026 WILEY.

**Figure 1:**
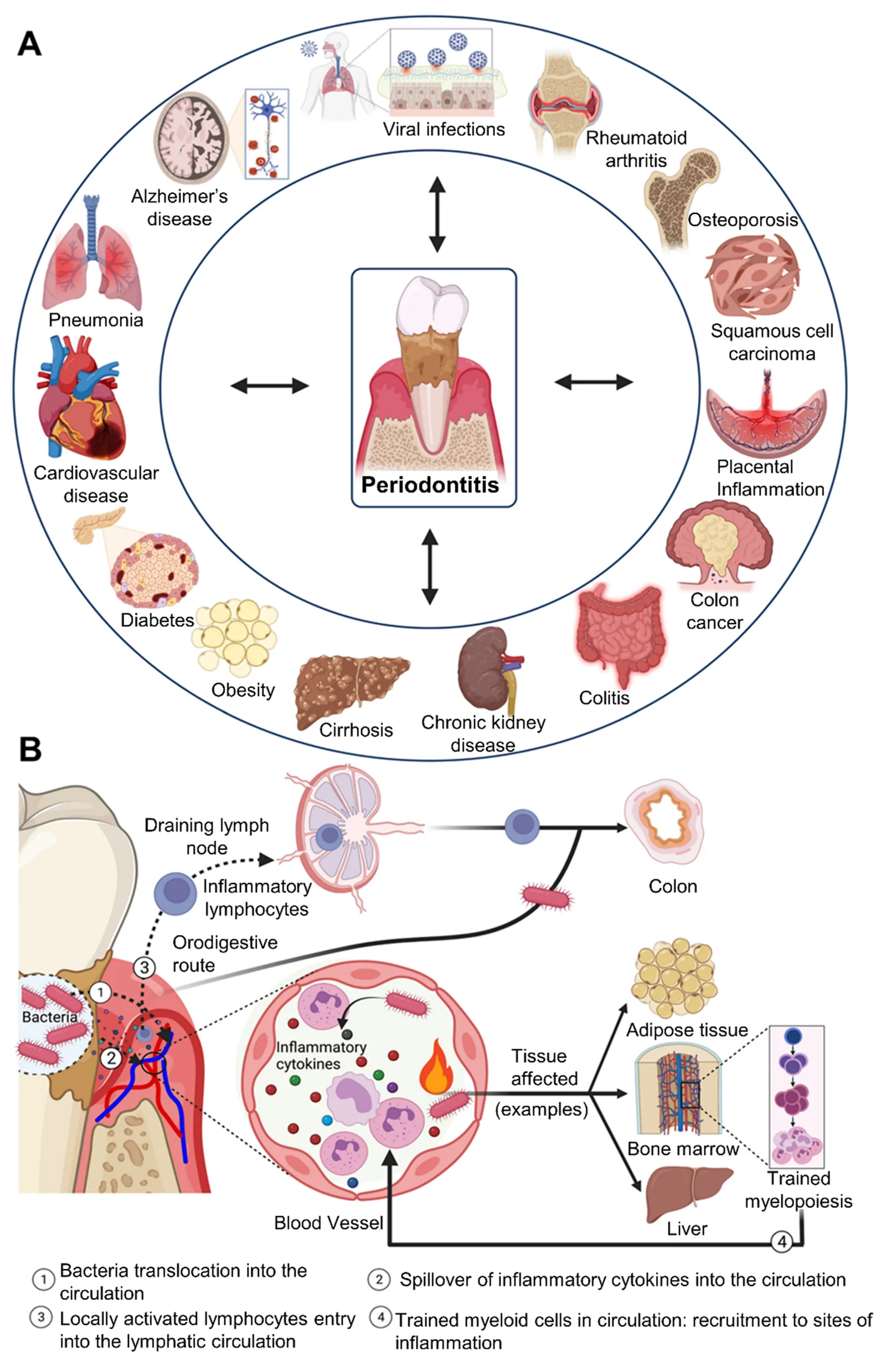
** (A-B)** Comorbidities with periodontitis. Possible pathways linking systemic inflammation and periodontitis. Adapted with permission from ref. 98, Copyrights 2022 WILEY.

**Figure 2:**
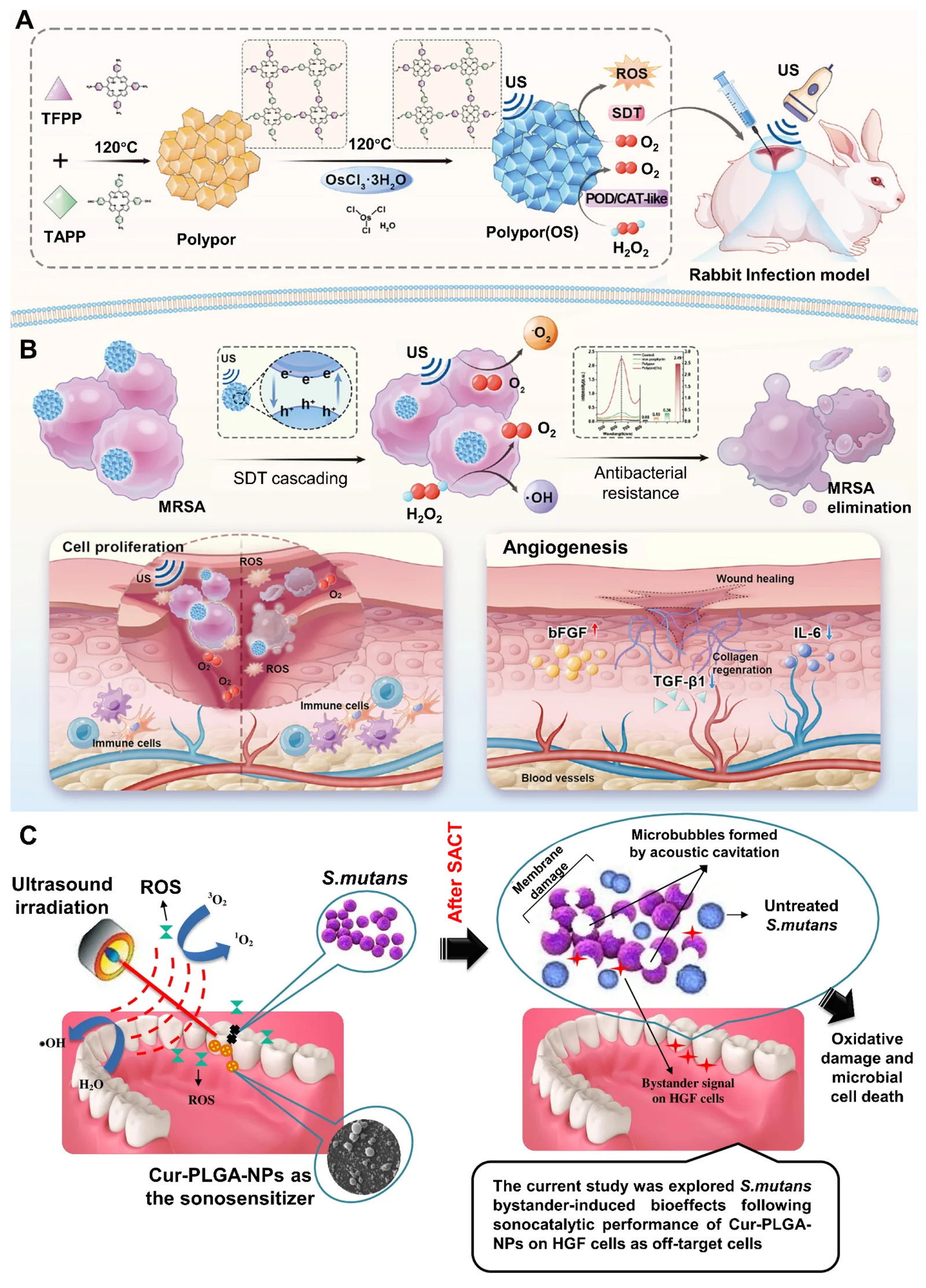
Engineered Polypor (Os) using a bioinspired strategy to promote wound healing by combining peroxidase (POD)-like catalysis, catalase (CAT)-mimetic activity, and ultrasound (US)-triggered ROS generation. **(A)** The schematic shows how researchers prepare Polypor (Os). **(B)** The illustration explains how ultrasound-driven ROS therapy promotes the healing of infected wounds through coordinated therapeutic pathways. Adapted with permission from ref. 103 Copyrights 2025, Wiley. **(C)** The figure depicts sonodynamic antimicrobial chemotherapy (SACT)-induced bystander effects in human gingival fibroblast (HGF) cells, which function as off-target cells. Adapted with permission from ref. 104, Copyrights 2020 ELSEVIER.

**Figure 3:**
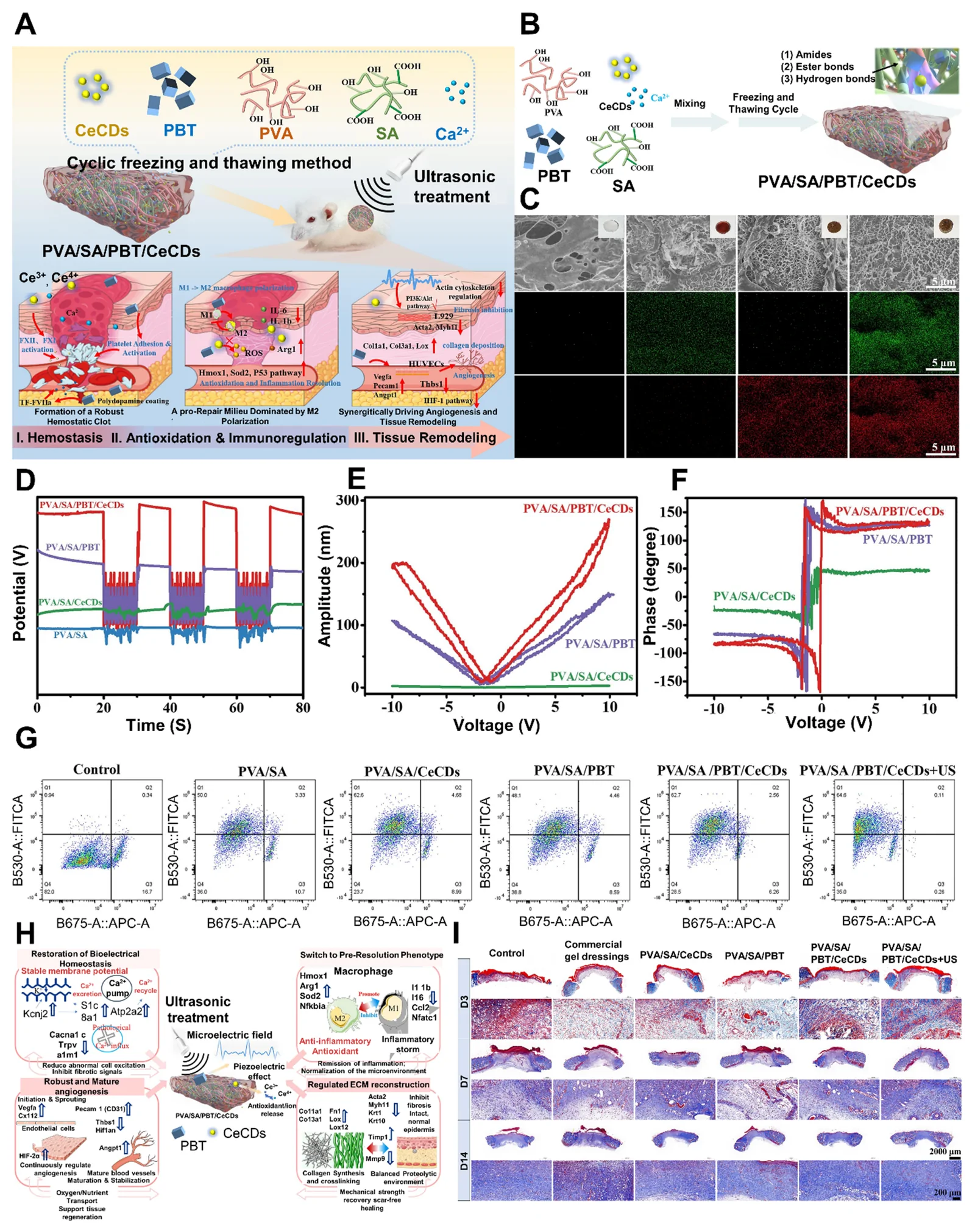
** (A-B)** Schematic illustration showing the hemostasis process, wound healing progression, and the fabrication route of the PVA/SA/PBT/CeCDs hydrogel. **(C)** Scanning electron microscopy (SEM) image of the hydrogel along with energy-dispersive X-ray spectroscopy (EDS) elemental mapping; the inset shows a macroscopic view (scale bar: 5 μm). **(D-F)** Electrochemical and mechanical efficiency during ultrasonic stimulation, exhibiting open-circuit voltage output, ferroelectric polarisation agitation, and the typical butterfly-like amplitude-voltage response. (n = 3 per group; results displayed as mean ± SD; ***p < 0.001, **p < 0.01, *p < 0.05). **(G)** Flow cytometry evaluation reveals macrophage polarisation with CD86⁺ (M1) and CD206⁺ (M2) populations. (H) Transcriptional data is paired with a schematic layout to depict the potential biochemical processes involved in ultrasound-induced wound repair. (I) Masson's trichrome-stained histological slices of wound tissue taken on days 3, 7, and 14 (scale bars: 2000 μm and 200 μm). Adapted with permission from ref. 117, Copyrights 2026 ELSEVIER.

**Figure 4:**
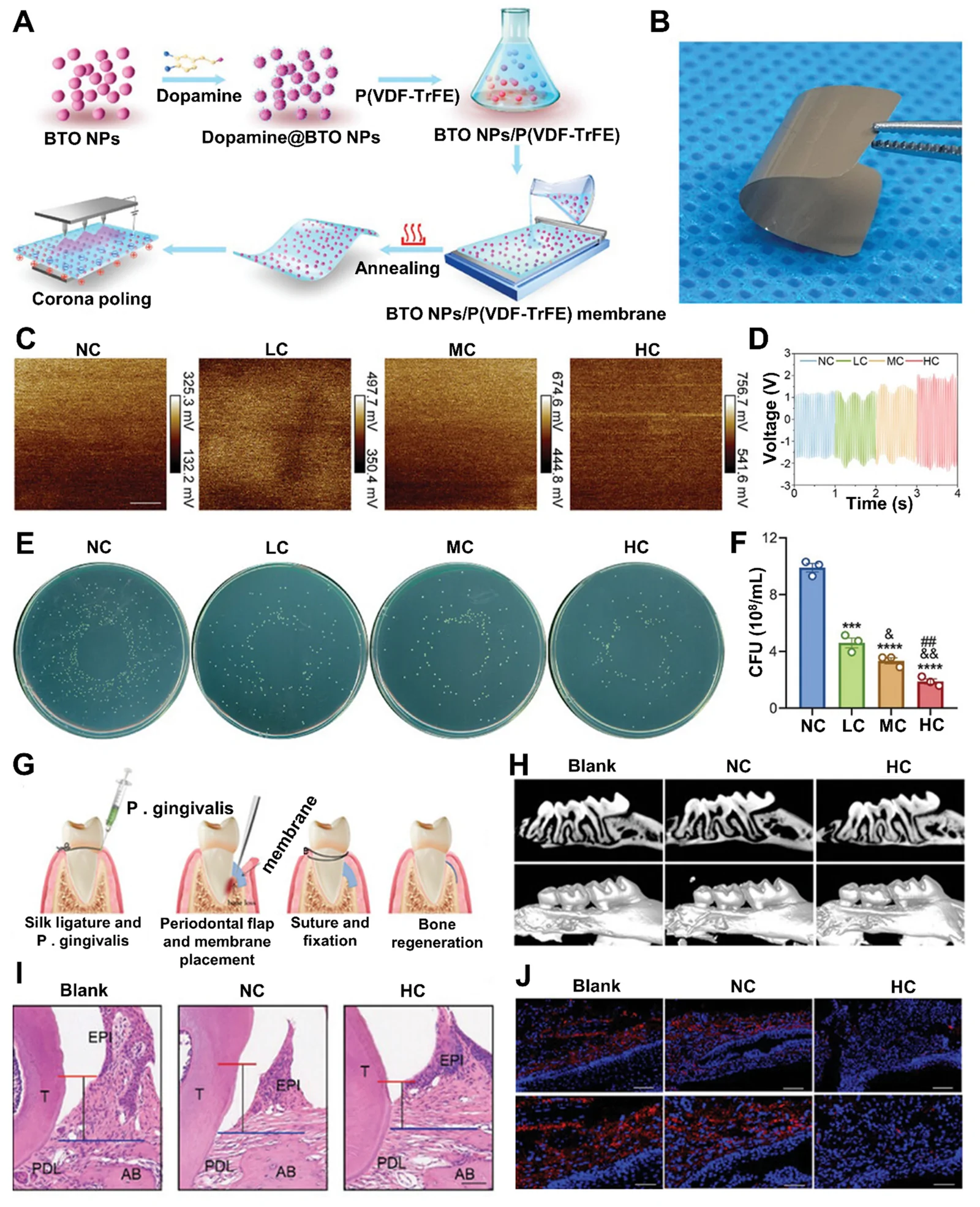
** (A)** Schematic illustration showing the fabrication process of BTO NP/P(VDF-TrFE) electroactive membranes (EMs) with different surface electrical potentials. **(B)** Photograph of the prepared BTO NP/P(VDF-TrFE) nanocomposite EMs. **(C)** Surface potential measurements of BTO NP/P(VDF-TrFE) EMs across different electrical polarization levels. **(D)** Output voltage performance of the BTO NP/P(VDF-TrFE) EMs under stimulation. **(E-F)** Representative images and quantitative analysis of *Staphylococcus aureus* colonies after co-culture with EMs exhibiting varying surface electrical potentials. **(G)** Schematic diagram illustrating the establishment of the *Porphyromonas gingivalis*-induced periodontitis model and the proposed therapeutic mechanism of the electroactive nanocomposite membrane. **(H)** Representative micro-CT sagittal views and 3D reconstructed images of maxillary alveolar bone around the second molar after 4 weeks of EM implantation with different surface charges. **(I)** Histological evaluation using H&E staining of maxillary alveolar bone surrounding the second molar after 4 weeks of treatment with different EM groups. **(J)** Fluorescence *in situ* hybridization (FISH) images showing detection of *P. gingivalis* around the second molar after 3 days of implantation of the various EMs. Adapted with permission from ref. 133, Copyrights 2024 WILEY.

**Figure 5:**
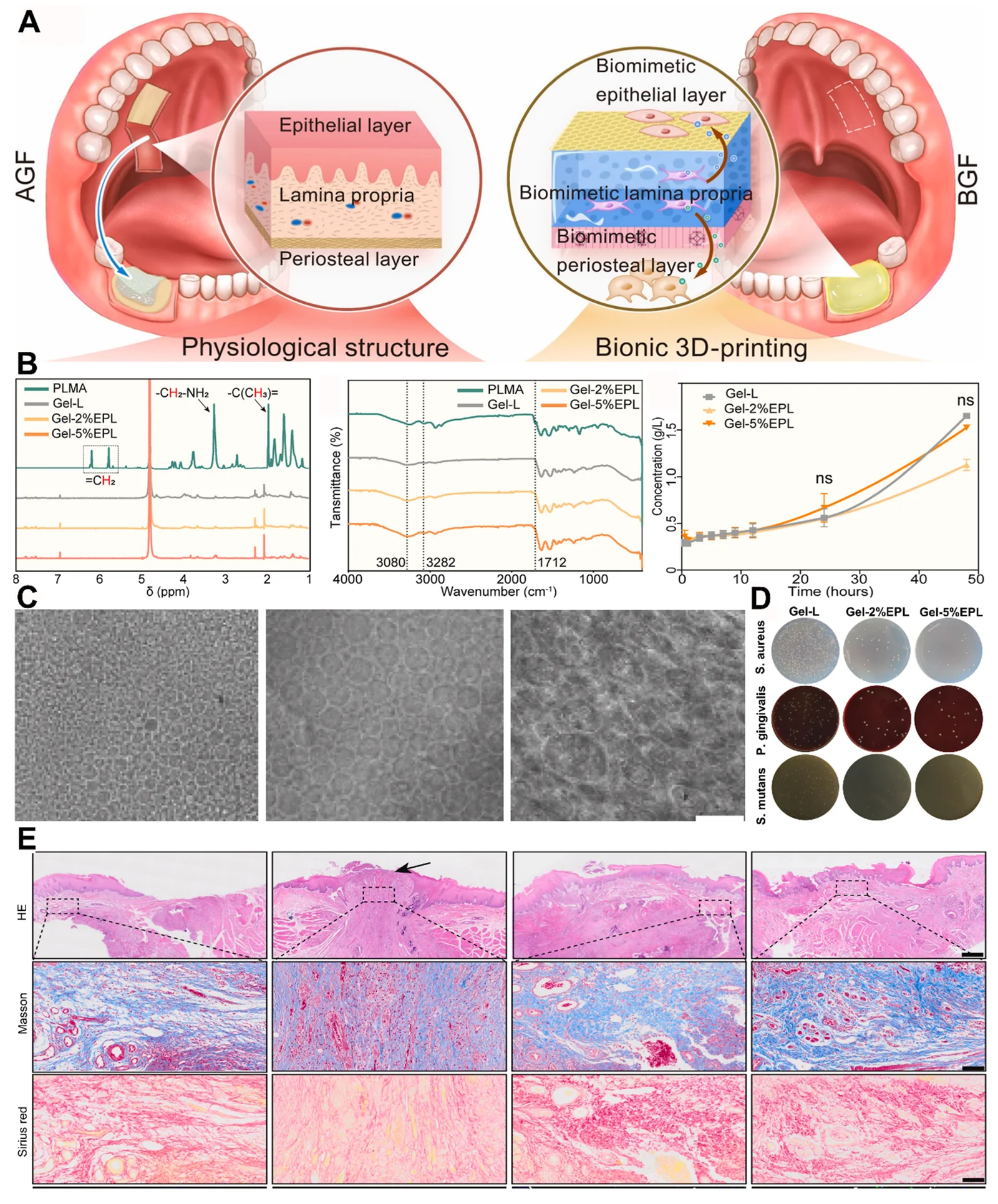
** (A)** Schematic illustration showing the biomimetic design strategy of the BGF system. **(B)** Characterization of PLMA, Gel-L, and Gel-EPL using ¹H NMR (D_2_O) and FTIR analysis, along with peptide release profiles of Gel-L and Gel-EPL; all experiments include three independent replicates (n = 3 per group), and “ns” indicates no statistical significance. **(C)** An optical microscopy image capturing the shape and structure of emulsion droplets within Gel-PEO (scale bar: 50 μm). **(D)** Antibacterial testing of Gel-EPL performed *in vitro*, shown through representative culture plate images of *Staphylococcus aureus*, *Porphyromonas gingivalis*, and *Streptococcus mutans* colonies following exposure to Gel-L versus Gel-EPL. **(E)** The evaluation of histologically regenerated rabbit soft tissue at 14 days using Masson's trichrome, Sirius Red, and H&E staining in the various experimental groups. The dashed box represents the region chosen for Sirius Red and Masson evaluation, while arrows indicate areas of considerable inflammatory cell proliferation (scale bar: 500 μm). Adapted with permission from ref. 144, Copyright 2026, ELSEVIER.

**Figure 6:**
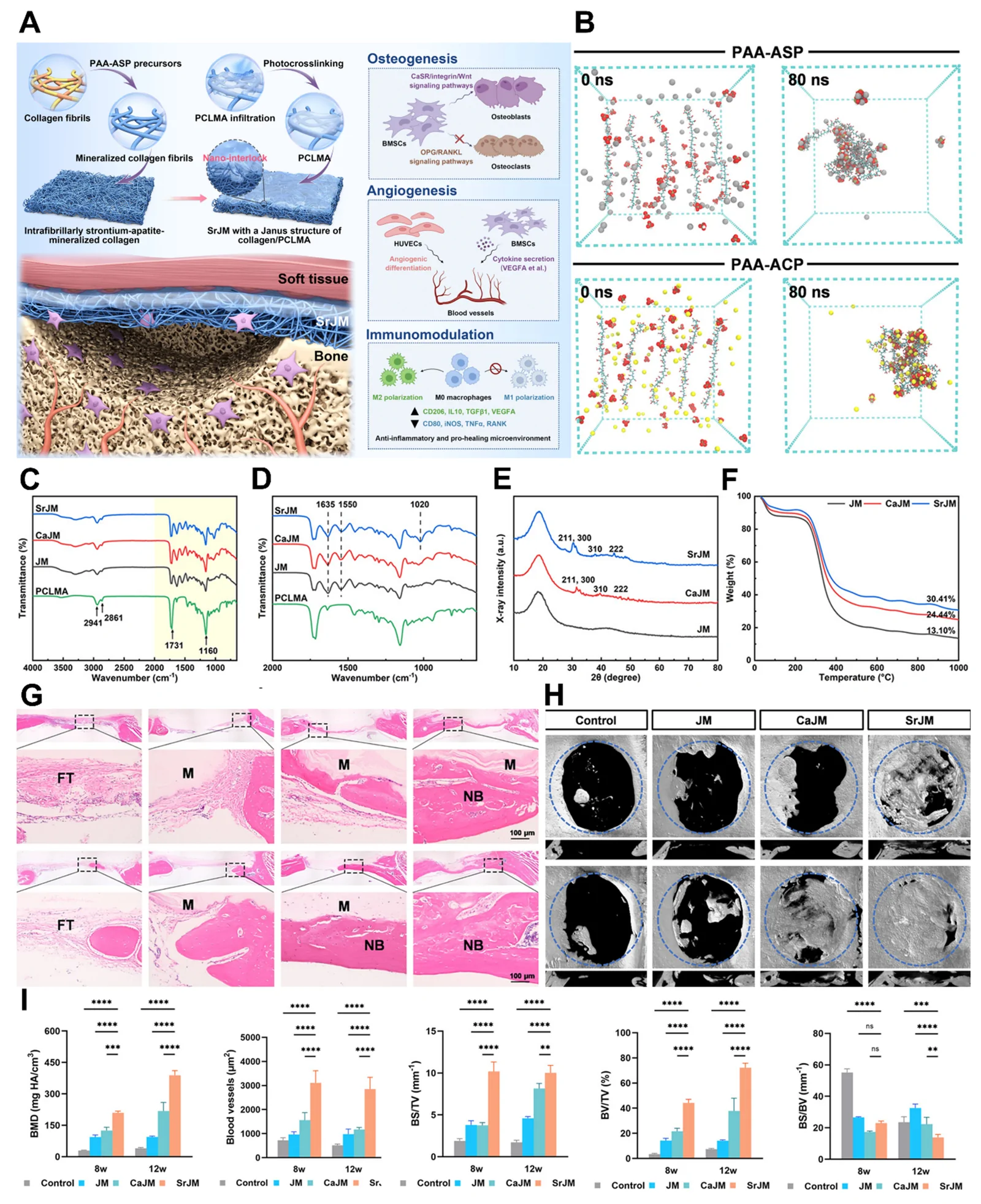
** (A)** Janus membrane production, application, and bioactive properties. **(B)** The molecular dynamics simulations illustrating the synthesis of PAA-ASP and PAA-ACP. **(C-F)** Janus membrane FTIR spectra, XRD analysis, and thermogravimetric assessment. **(G-H)** H&E staining using fragmented and reconstituted micro-CT pictures taken eight and twelve weeks after implantation. **(I)** Fluorescence intensity, BMD, BS/TV, BV/TV, and BS/BV are quantitative micro-CT measurements. Adapted with permission from ref. 162, Copyright 2024, AMERICAN CHEMICAL SOCIETY.

**Figure 7:**
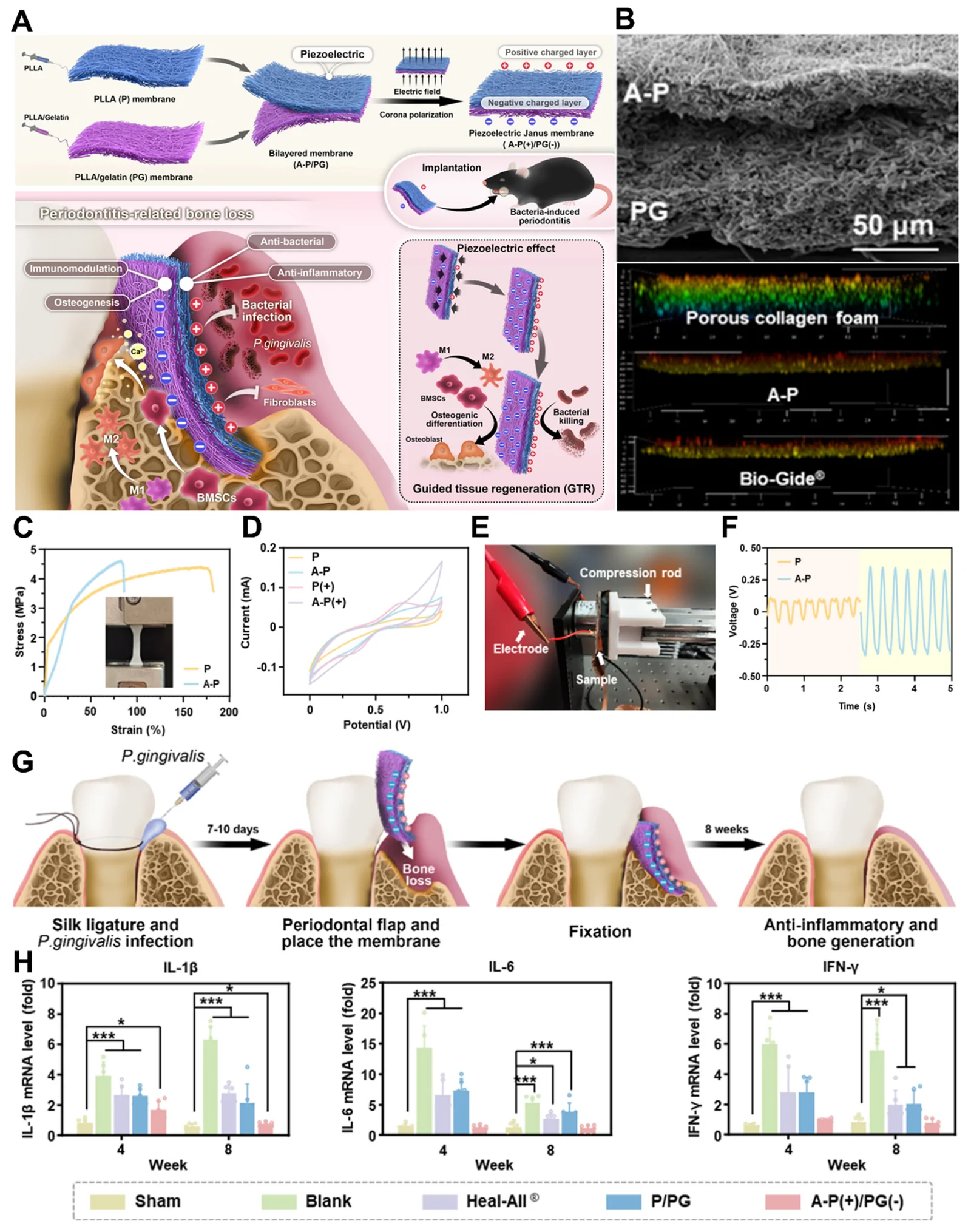
** (A)** Design and Development of a Biodegradable Piezoelectric Janus Membrane for Bone Regeneration and Antibacterial Therapy in Periodontitis. **(B)** SEM images of the bilayered A-P/PG membrane's cross section and CLSM photographs of fibroblasts penetrating collagen foam while being stopped by the A-P and Bio-Gide membrane. **(C)** P and A-P membrane tensile stress-strain curves. The test setup is shown in the inset. **(D)** PLLA membrane C-V curves**. (E)** An image of the electrometer used to measure the membranes' piezoelectric output performance. **(F)** A-P membrane's piezoelectric output performance under different pressures. A comparison of P and A-P membranes' piezoelectric output performance. **(G)** An illustration of the ligation process used to create a mouse model of periodontitis. The maxillary second molars' cervical region was bound with a ligature on both sides. **(H)** RT-qPCR to measure the expression levels of three inflammatory genes (IL-1β, IL-6, and INF-γ). Adapted with permission from ref. 171, Copyrights 2025, AMERICAN CHEMICAL SOCIETY.

**Figure 8:**
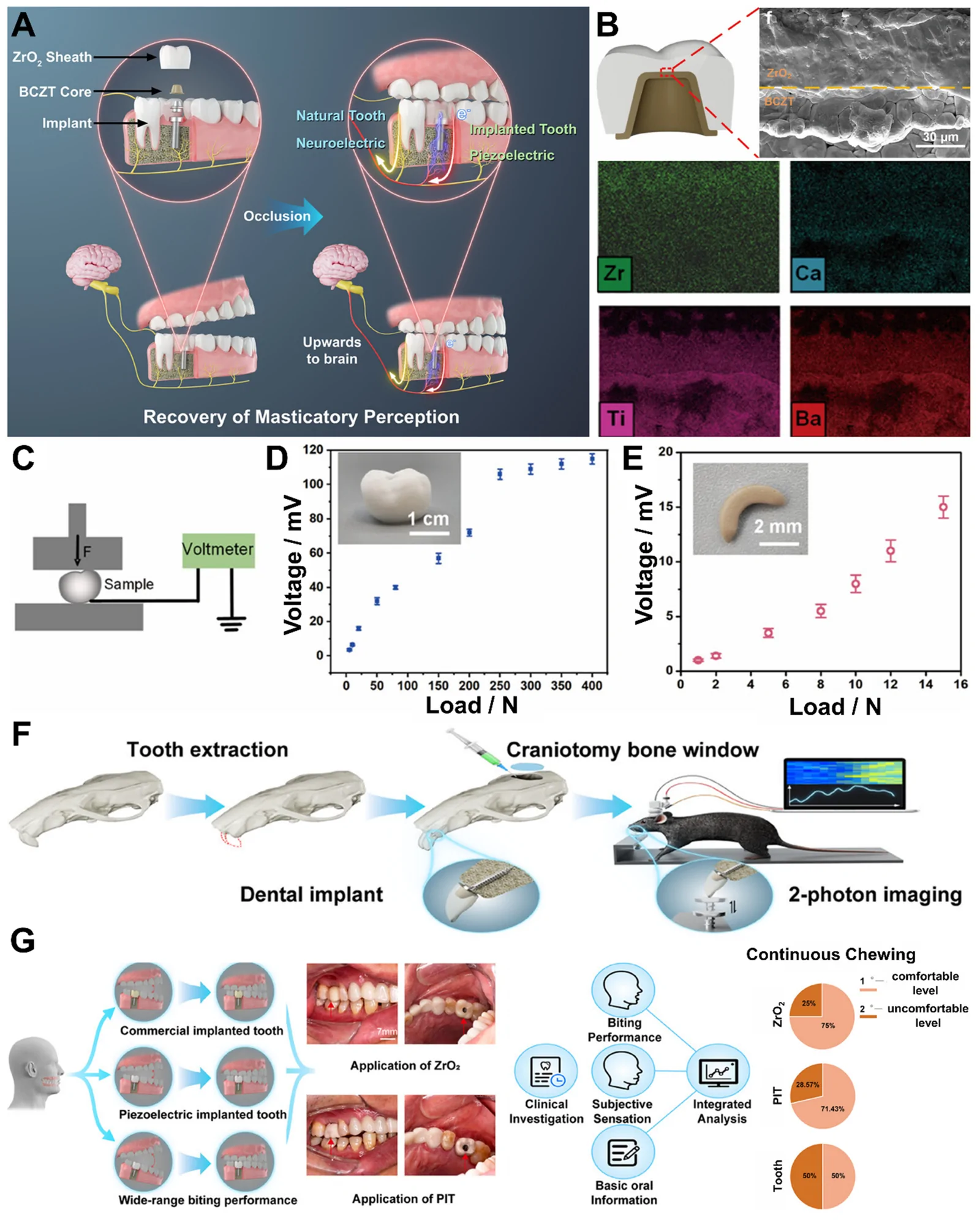
** (A)** An exploded image of a PIT during dental implantation is shown schematically. Patients' masticatory perception skills can be restored by the PIT, allowing them to sense food contact in their mouths. **(B)** SEM cross-section of the BCZT scaffold. **(C-E)** Diagrammatic depiction of one-way piezoelectric tester probing, the PIT's output voltage levels for humans and mice are dependent on the applied pressures. **(F)** Schematic depiction of dental implantation and picture capture processes. **(G)** Clinical Application of perceptive teeth to restore occlusal feeling, representation of a clinical masticatory sensory test utilizing a normal tooth, PIT dental crown, and ZrO_2_ dental crown. Red arrows show the PIT test and the degree of response of patients wearing normal teeth, ZrO_2_ dental crowns, and PIT dental crowns during continuous chewing. Adapted with permission from ref. 178, Copyrights 2026 WILEY.

**Figure 9:**
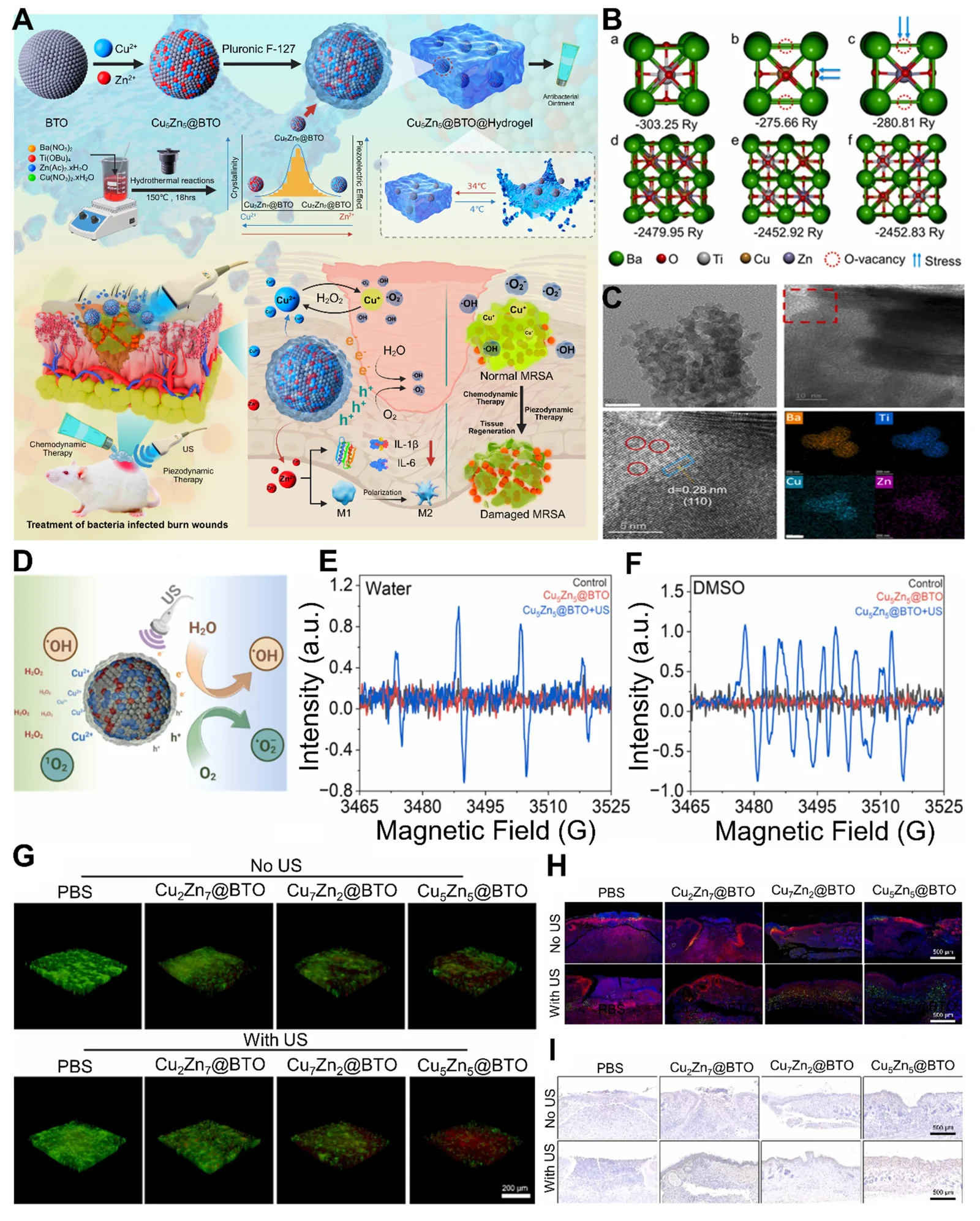
** (A)** shows a schematic of the production and gelation process of the piezoelectric hydrogel (Cu_5_Zn_5_@BTO), followed by an illustration of the mechanism of wound healing, in which PZDT contributes to antibacterial action, Zn^2+^ ions provide anti-inflammatory effects, and Cu^2+^ ions drive CDT. **(B)** To confirm the microstructural characteristics and pinpoint the source of the material's piezoelectric behaviour, first-principles computational analysis was employed. **(C)** Cu_5_Zn_5_@BTO transmission electron microscopy (TEM) pictures showing the surface morphology (size bar = 20 nm). High-resolution TEM images show short-range structural disorder (scale bar = 5 nm) by highlighting localised lattice faults (shown by red circles) and the associated (110) crystal plane. The elemental distribution inside Cu_5_Zn_5_@BTO is confirmed by energy-dispersive spectroscopy (EDS) mapping (scale bar = 200 nm). **(D)** A schematic representation of the developed system's ROS generating process. Cu_5_Zn_5_@BTO's electron paramagnetic resonance (EPR) spectra, which employ DMPO as the trapping agent and show **(E)** •OH creation in water and **(F)** •O₂⁻ generation in DMSO under ultrasonic stimulation, reveal the production of ROS. **(G)** Three-dimensional confocal laser scanning microscopy (CLSM) pictures of bacterial biofilms following various treatments employing Live/Dead staining, where red fluorescence denotes dead bacteria and green fluorescence shows living bacteria. **(H)** Immunofluorescence labelling of wound tissue sections with iNOS, Cy3, ARG1, and FITC markers reveals an increase in ARG1-positive (M2) macrophages and a reduction in iNOS-positive (M1) macrophages. **(I)** The Cu_5_Zn_5_@BTO+US group exhibits decreased expression of CD86 in immunohistochemical staining, which further supports the anti-inflammatory action. Adapted with permission from ref. 186, Copyrights 2025 ELSEVIER.

**Figure 10:**
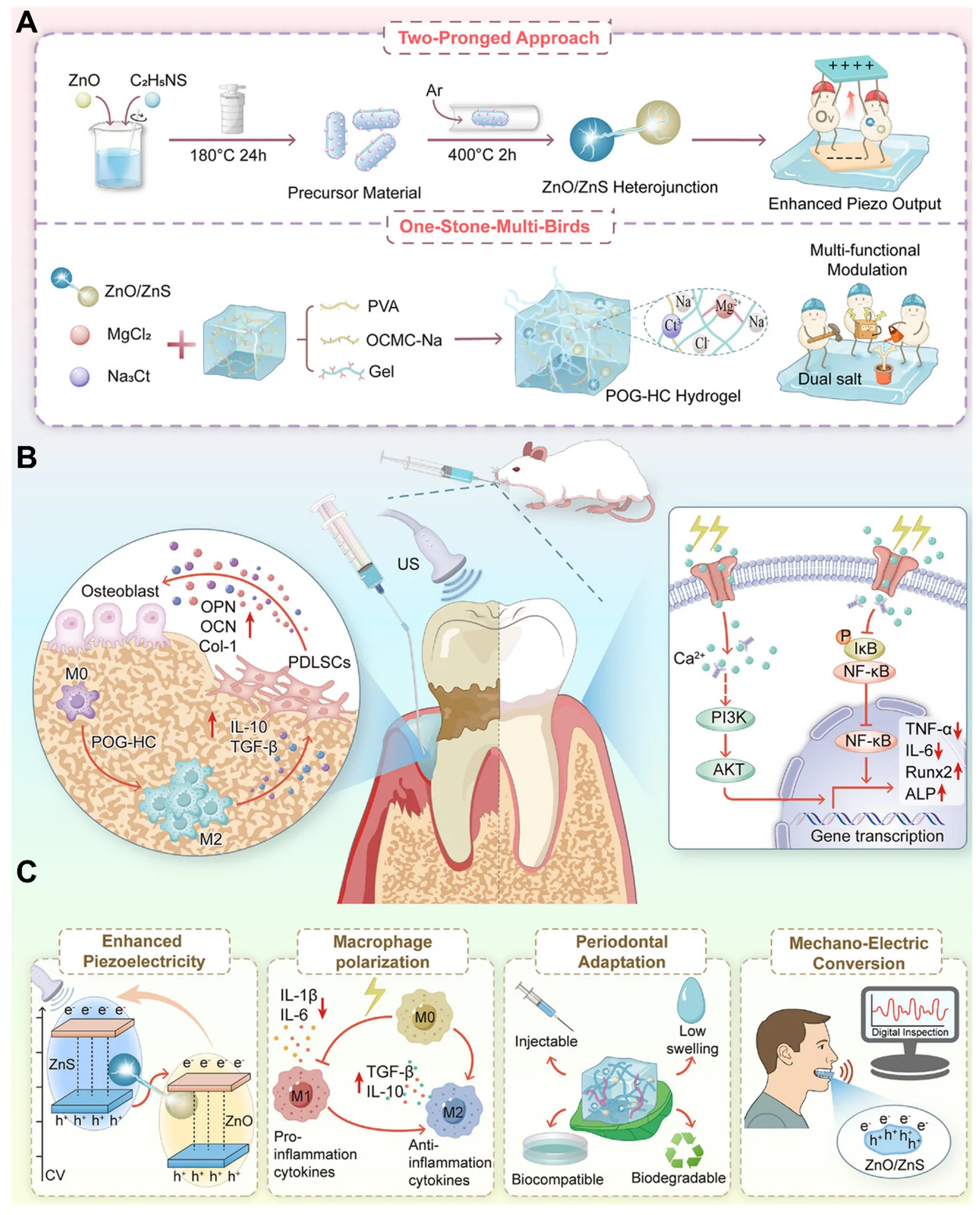
Piezoelectric hydrogel system designed for periodontal therapy with an integrated dual-strategy framework. **(A)** Dual-enhancement concept: the combination of heterojunction formation and oxygen vacancy engineering improves the piezoelectric performance of ZnO. Multi-functional strategy: incorporation of dual salts (MgCl_2_/Na_3_Ct) strengthens the hydrogel by improving mechanical properties, ionic conductivity, and osteoinductive transport. **(B, C)** The resulting hydrogel system delivers enhanced piezoelectric output, regulates immune responses, and maintains tissue adaptability, thereby supporting coordinated osteoimmune regeneration. Adapted with permission from ref. 199, Copyrights 2026 WILEY.

**Figure 11:**
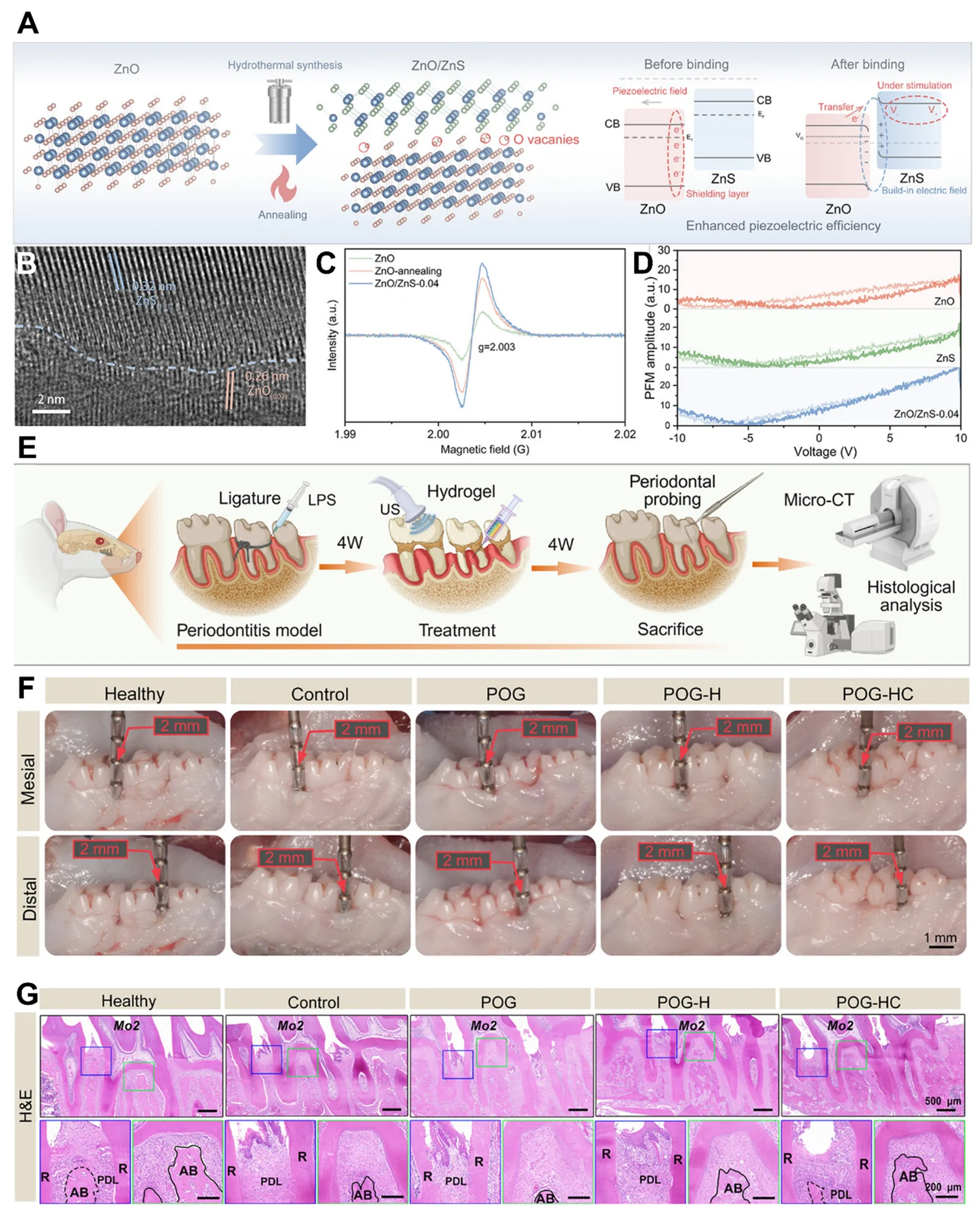
** (A)** The process of the fabrication of ZnO/ZnS heterostructure and the piezoelectric enhancement mechanism.** (B)** High-resolution TEM image of ZnO/ZnS-0.04 with clearly resolved lattice fringes of ZnO and ZnS indicating the presence of heterojunctions (scale bar: 2 nm). **(C)** The electron paramagnetic resonance (EPR) spectra of pure ZnO, annealed and ZnO/ZnS-0.04 is shown. **(D)** A comparison of piezoelectric response in pure ZnO, pure ZnS and ZnO/ZnS-0.04 using Piezoresponse force microscopy (PFM) curves. **(E)** Experimental model of periodontitis created in rats by placing ligatures, injecting LPS, and treating with piezoelectric hydrogel. **(F)** Periodontal probing measurements on the palatal side of the rats' maxillary second molars in mesial and distal locations (n = 4; scale bar: 1 mm). **(G)** Histological evaluation of the periodontal ligament (PDL) and alveolar bone (AB); H&E staining showed that the root (R) was surrounded by the PDL (n = 4; scale bars: 500 µm/200 µm); TRAP staining involved red staining areas, which indicated osteoclast activity, and the second molar (Mo2) was used as a control. Adapted with permission from ref. 199, Copyright 2026, WILEY.

**Figure 12:**
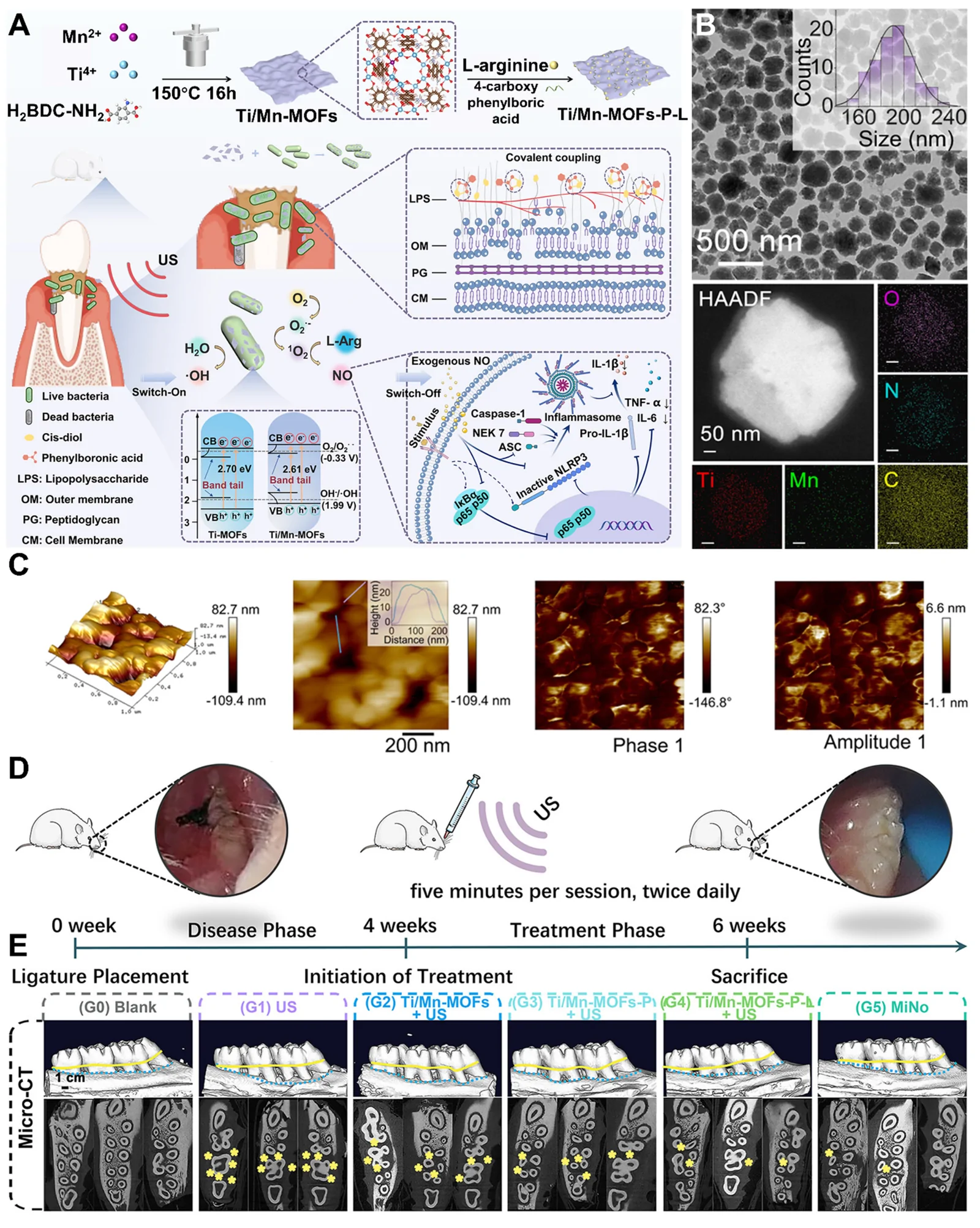
** (A) Schematic illustration of the development and mechanism of Ti/Mn-MOFs-P-L in periodontitis treatment. (B) Size distribution, TEM image and elemental mapping of Ti/Mn-MOF and HAADF-STEM characterization. (C) Ti/Mn-MOF PFM characterization including 2D and 3D out-of-plane PFM, phase picture and amplitude image. (D)** The* in vivo* experimental schematic design. (E) Micro-CT scans of MOF. Adapted with permission from ref. 210, Copyrights 2025 AMERICAN CHEMICAL SOCIETY.

**Figure 13:**
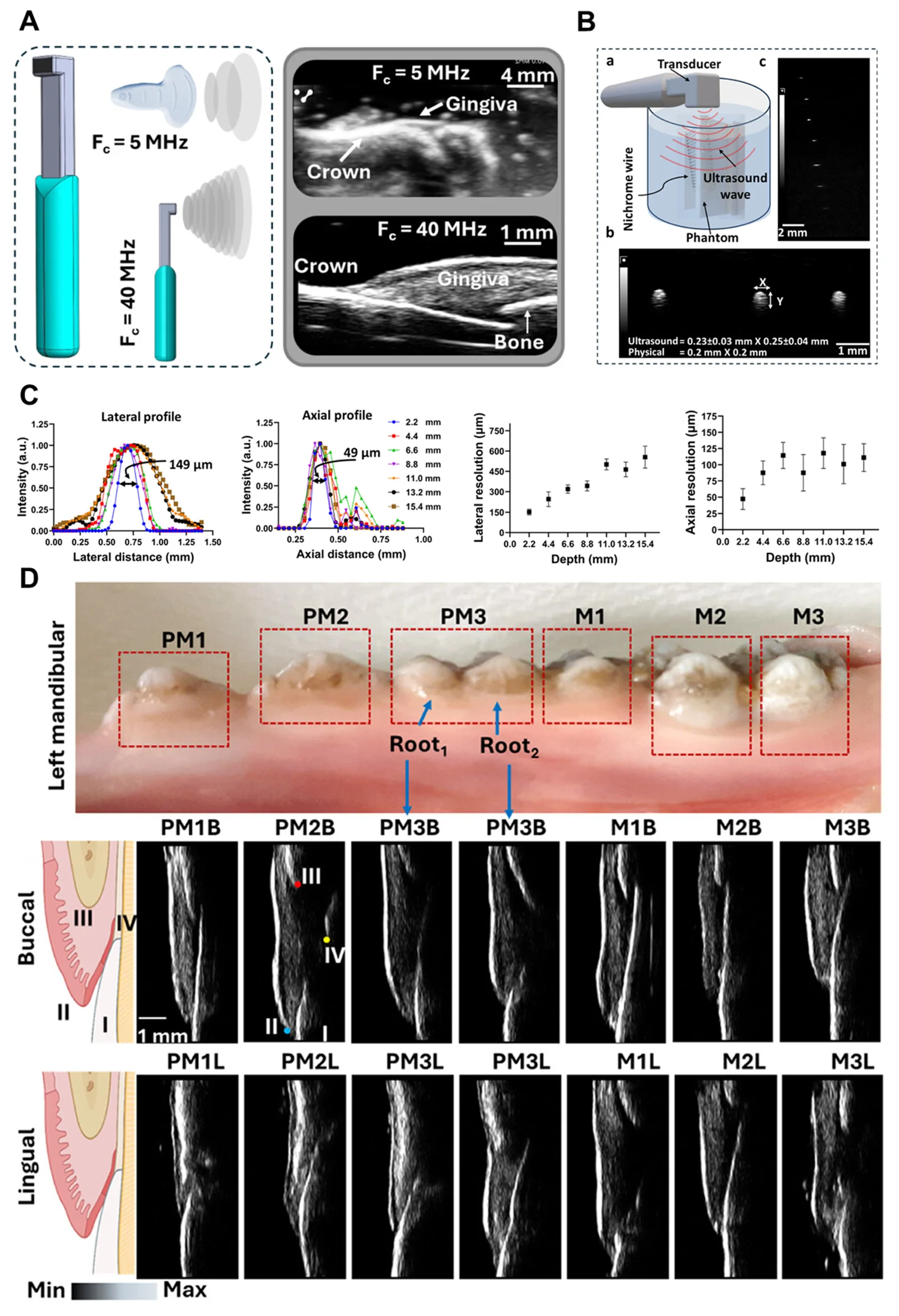
** (A)** High frequency periodontal transducer with a toothbrush-like shape, and a schematic of a nichrome wire phantom immersed in water. **(B)** An ultrasound image of a pencil-lead phantom to compare the measured diameter to the actual diameter of the pencil and ultrasound images of several different nichrome wires at different depths. The averaged lateral resolution was 149 ± 21 μm, and the averaged axial resolution was 49 ± 15 μm over the measured depth range of 2.2 mm to 15.4 mm with **(C)** line spread function data for a nichrome wire. **(D)** An excised swine mandible was *in vitro* imaged. The jaw has a photograph attached that shows three molars (M1-M3) and three premolars (PM1-PM3) remaining in place. The ultrasound scans made from buccal and lingual sides revealed clearly the important anatomical features I (Crown), II (GM), III (ABC), and IV (CEJ). Reprinted from ref. 225 with permission from the American Chemical Society.

**Table 1:** Summary of standard materials that are responsive to ultrasound.

Material System	Responsiveness	Mechanism Type	Application	References
TSPBA hydrogel system	US	Borate bond cleavage	Periodontitis	54
PBA-HA implant coating	US	ROS-triggered degradation	Peri-implant inflammation	55
CAPE-chrysin PLGA hydrogel system	US	ROS-sensitive release system	Periodontitis	56
Oxidized dextran hydrogel system	US	Oxidation-triggered release	Periodontitis	57
COF-based system	US	Oxidation-responsive COF	Periodontitis	58
PLGA microneedle system	US	Thioketal bond cleavage	Periodontitis	59
Polypyrrole hydrogel system	US	Conductive stimulation	Peri-implant repair	60
BTO hydrogel system	US	Piezoelectric effect	Periodontitis	61
BTO-gelatin system	US	Piezoelectric stimulation	Periodontitis	62
ZnO/TiO_2_ heterojunction system	US	Piezoelectric stimulation	Periodontitis	63

**Table 2:** Mechanistic comparison of major ultrasound-responsive therapeutic modalities in periodontitis treatment.

Type	Energy Input	Physical Process	US Conditions	Primary Output	Periodontal Application	References
SDT	Ultrasound + sonosensitizer	Acoustic cavitation activates sonosensitizers	(0.5-3 MHz), moderate-intensity pulsed US; cavitation-dependent	ROS	Biofilm disruption, bacterial eradication, and suppression of periodontal inflammation	90
PZDT	Ultrasound + piezoelectric material	Ultrasound-induced piezoelectric polarization and charge separation	Low- to moderate-intensity US (0.5-5 MHz); mechanical deformation-driven	ROS + transient electrical charges	Simultaneous antibacterial therapy and periodontal tissue	94
Piezocatalysis	Mechanical stimulation (ultrasound, vibration, fluid flow)	Piezoelectric surface polarization drives redox reactions	Continuous or pulsed mechanical/ US stimulation; catalyst-dependent	ROS	Elimination of persistent periodontal pathogens and biofilm control	93
Piezoelectric Bioelectrical Stimulation	Mechanical loading or low-intensity ultrasound	Piezoelectric conversion of mechanical energy into localized bioelectrical signals	Low-intensity stimulation with minimal cavitation and thermal effects	Electrical cues	Periodontal ligament regeneration, osteogenesis, and alveolar bone reconstruction	92
